# A catalogue of recombination coldspots in interspecific tomato hybrids

**DOI:** 10.1371/journal.pgen.1011336

**Published:** 2024-07-01

**Authors:** Roven Rommel Fuentes, Ronald Nieuwenhuis, Jihed Chouaref, Thamara Hesselink, Willem van Dooijeweert, Hetty C. van den Broeck, Elio Schijlen, Henk J. Schouten, Yuling Bai, Paul Fransz, Maike Stam, Hans de Jong, Sara Diaz Trivino, Dick de Ridder, Aalt D. J. van Dijk, Sander A. Peters

**Affiliations:** 1 Bioinformatics Group, Wageningen University and Research, Wageningen, The Netherlands; 2 Chromosome Biology, Max Planck Institute for Plant Breeding Research, Cologne, Germany; 3 Business Unit of Bioscience, Cluster Applied Bioinformatics, Wageningen University and Research, Wageningen, The Netherlands; 4 Swammerdam Institute for Life Sciences, University of Amsterdam, Amsterdam, The Netherlands; 5 Department of Human Genetics, Leiden University Medical Center, Leiden, The Netherlands; 6 Centre for Genetic Resources, Wageningen University and Research, Wageningen, The Netherlands; 7 Plant Breeding, Wageningen University and Research, Wageningen, The Netherlands; 8 Laboratory of Genetics, Wageningen University and Research, Wageningen, The Netherlands; Max Planck Institute of Molecular Plant Physiology: Max-Planck-Institut fur molekulare Pflanzenphysiologie, GERMANY

## Abstract

Increasing natural resistance and resilience in plants is key for ensuring food security within a changing climate. Breeders improve these traits by crossing cultivars with their wild relatives and introgressing specific alleles through meiotic recombination. However, some genomic regions are devoid of recombination especially in crosses between divergent genomes, limiting the combinations of desirable alleles. Here, we used pooled-pollen sequencing to build a map of recombinant and non-recombinant regions between tomato and five wild relatives commonly used for introgressive tomato breeding. We detected hybrid-specific recombination coldspots that underscore the role of structural variations in modifying recombination patterns and maintaining genetic linkage in interspecific crosses. Crossover regions and coldspots show strong association with specific TE superfamilies exhibiting differentially accessible chromatin between somatic and meiotic cells. About two-thirds of the genome are conserved coldspots, located mostly in the pericentromeres and enriched with retrotransposons. The coldspots also harbor genes associated with agronomic traits and stress resistance, revealing undesired consequences of linkage drag and possible barriers to breeding. We presented examples of linkage drag that can potentially be resolved by pairing tomato with other wild species. Overall, this catalogue will help breeders better understand crossover localization and make informed decisions on generating new tomato varieties.

## Author summary

Ensuring a stable food supply in a changing climate hinges on enhancing plants’ natural defenses and resilience. Breeders achieve this by crossing cultivated plants with their wild counterparts, mixing specific gene variants through genetic recombination. Yet, in some cases, certain genomic areas lack this recombination, particularly when crossing diverse plant types, limiting the mix of beneficial genes. In this study we used a method called pooled-pollen sequencing to map regions where genetic recombination does and doesn’t occur when breeding tomatoes with five wild relatives. We discovered specific regions where genetic exchange is rare, mainly due to structural differences in DNA. These regions tend to be rich in repetitive DNA sequences and are less likely to swap genes during reproduction. Importantly, many of these "recombination coldspots" contain genes related to plant health and stress resistance, inadvertently limiting the effectiveness of breeding efforts. By identifying these regions, breeders can now make more informed choices when developing new tomato varieties, potentially by incorporating genes from other wild species to overcome these limitations and improve crop resilience.

## Introduction

Crop breeding relies on the availability of genetic diversity to generate novel allele combinations that are agronomically valuable. However, long term selection by inbreeding often causes loss of essential genetic variation. To reintroduce lost alleles, breeders introgress new genetic material by crossing crops with wild relatives, followed by repeated backcrossing and selection. Among the most desirable traits to be incorporated into the breeding material are abiotic stress tolerance and disease resistance, yield, and fruit quality [1]. The success of introgression breeding largely depends on the process of meiotic recombination to introduce genetic material from the donor into the recipient crop [2]. Meiotic recombination, commonly referred to as crossover (CO), facilitates the exchange of chromosomal segment between parental chromosomes, shuffling alleles to make new combinations. Lack or even complete absence of recombination in a genomic region leads to linkage drag, i.e. the introgression of deleterious alleles along with the beneficial one. This can severely limit the ability to develop novel desired allele combinations. Chromosome regions where recombination is suppressed are found in pericentromeres (PER), including retrotransposons and other DNA-methylated regions [3,4]. Moreover, genomic rearrangements, in particular structural variants (SVs), affect recombination patterns, especially in hybrids [5–8].

Genomic rearrangements may exist between related species and between different genotypes of the same species and can lead to recombination coldspots, some of which are associated with resistance genes or adaptive traits [9,10]. Due to absent or diminished crossovers in SV regions, clusters of tightly linked alleles known as supergenes are inherited together as a single locus, contributing to local adaptation and reproductive isolation [11–13]. Suppression or absence of recombination has been found essential in speciation and domestication by allowing the fixation of alleles [14,15]. In the backcross descendants of a *Solanum habrochaites* introgression into cultivated tomato (*S*. *lycopersicum*), an inversion containing the *Ty-2* resistance genes and at least 35 more genes causes linkage drag, rendering selection of desirable agronomic trait combinations in the offspring impossible [16,17]. The instances of CO suppression presented here underscore the importance of investigating the impact of genomic rearrangements on recombination patterns and overall crop adaptation. Addressing these challenges is crucial for enhancing the effectiveness of introgressive breeding strategies and ensuring the successful development of crops with desirable traits.

Considered as one of the most cultivated vegetables crop, tomato has become a model system for genetic, developmental and physiological studies of fleshy fruits and is among the most well-studied crops for meiosis [18–21]. The availability of at least 12 wild relative species of tomato [22] makes it ideal for the study of recombination patterning in relation to genetic features such as SVs. Although previous studies addressed the role of SVs as recombination barriers [6,23], a genome-wide analysis of decreased or absent COs related to SVs and other genome features in tomato and multiple hybrid crosses is currently lacking, due to the absence of cost-effective and high-resolution crossover detection methods and accurate SV prediction. In particular, it is currently unclear whether coldspots are conserved (i.e. occur in the same genomic region in various genotypes) or more specific. To better understand the occurrence of recombination coldspots, we profiled the recombination landscape in multiple crosses of tomato and wild relatives by sequencing pools of pollen gametes. We identified coldspots in each hybrid cross and related their occurrence to genomic features. Our results suggest a major role for SVs and transposable elements in shaping the recombination landscape in hybrids, specifically in suppressing COs in a group of linked genes that relate to adaptation, speciation, and domestication. Finally, we show examples on how this catalogue can help determine bottlenecks in tomato introgressive hybridization breeding.

## Results

### Crossovers in multiple hybrid crosses

**Fig 1:**
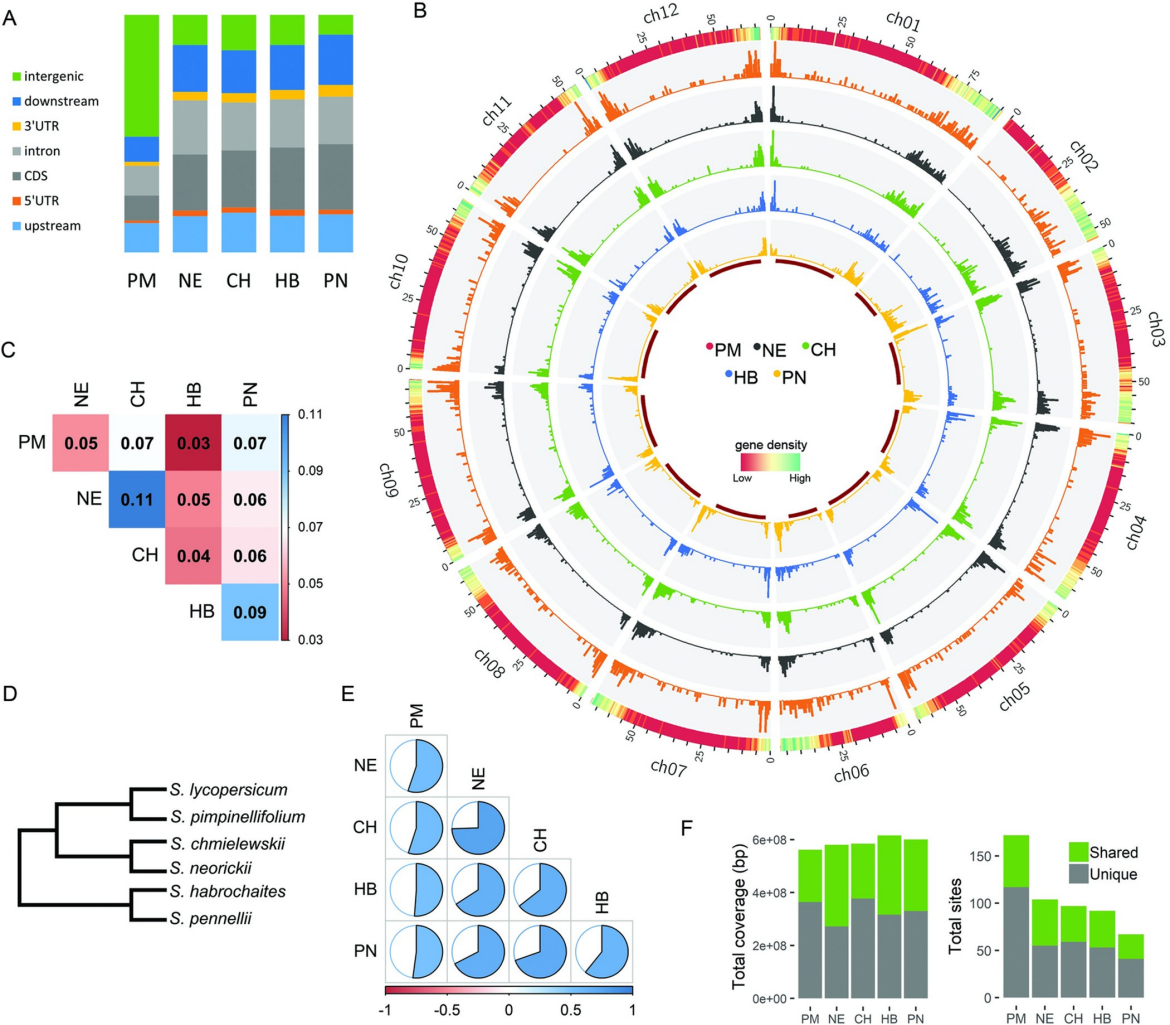
Divergent recombination landscapes. A) Distribution of crossover regions over gene features. Upstream and downstream covers 1 kb from the transcription start and termination sites, respectively. B) Distribution of COs per hybrid. Y-axis shows the number of COs per 500-kb window. The outermost track indicates gene density while the red innermost track marks the pericentromeric regions. C) Fraction of shared CO sites between hybrids. D) Phylogenetic tree of the parental species based on Moyle [22]. E) Correlation of the genome-wide CO landscape between hybrids (all P < 2.2 x 10^−16^). F) Coverage and number of recombination coldspots in different crosses.

We have generated hybrid crosses of *S*. *lycopersicum Heinz1706* and its wild relatives *S*. *pimpinellifolium* (CGN14498; **PM**), *S*. *neorickii* (LA0735; **NE**), *S*. *chmielewskii* (LA2663; **CH**), *S*. *habrochaites* (LYC4; **HB**), and *S*. *pennellii* (LA0716; **PN**). Hereafter, we use these abbreviations and the species name when referring to the hybrids and the parental genome, respectively. The pool of pollen from each hybrid was sequenced using 10X Genomics kits (**S1 Table**) based on the protocol described in Fuentes, et al. [24]. We identified recombinant molecules and reported a total of 6,382 COs in all hybrids, primarily located in distal segments of chromosomes (**S2 Table and S1 Fig and S1 Text**), consistent with previous reports in tomato and other plant species [3,8]. CO regions account for only 2% of the whole genome (relative to SL4.0), matching observations in other eukaryotic organisms where recombinations are concentrated in hotspots [3,8,25,26]. The vast majority (5,150; 81%) of COs are located within genes and their 1kb flanking regions, while another 471 are positioned between 1kb and 3kb from genes (**Table 1** and **Figs 1A** and S2). Despite known low recombination rates in tomato pericentromeres [27,28], we detected there a total of 710 COs (11.1%) in all hybrids. These are likely located in euchromatin islands within the PER. It has been proposed that the suppression of double-strand-breaks (DSBs), precursors of COs, by condensed repeat-rich chromatin like PER helps safeguard against genome destabilization [28,29]. Apart from enrichment in genic regions, our results thus show that some COs occur in PER, unlike previously assumed.

**Table 1:** Crossovers detected in multiple interspecific (with *S*. *lycopersicum*) hybrid populations.

Hybrid Cross	Number of SNPs	Number of COs	Distance (kb; 1/Resolution)	Distal euchromatin genes (*p*-val) *	Pericentromere genes (*p*-val)*
***S*. *pimpinellifolium* (PM)**	4,742,049	1,040	2.3 ± 1.4	9.3 x 10^−3^	8.2 x 10^−5^
***S*. *neorickii* (NE)**	13,749,445	1,700	2.3 ± 1.5	2.1 x 10^−107^	4.3 x 10^−20^
***S*. *chmielewskii* (CH)**	13,770,207	1,618	2.2 ± 1.5	2.3 x 10^−104^	4.4 x 10^−16^
***S*. *habrochaites* (HB)**	14,909,955	832	1.9 ± 1.5	6.9 x 10^−66^	1.5 x 10^−9^
***S*. *pennellii* (PN)**	15,447,841	1,192	2.1 ± 1.6	1.2 x 10^−86^	7.8 x 10^−30^

*Enrichment of COs in genes based on permutation test

### Unique recombination patterns between hybrids

All hybrids show similar recombination landscapes, with COs mostly in distal, gene-rich chromosome regions. Yet there are distinct local patterns of COs, as illustrated in **Fig 1B**. Comparisons of recombination profiles among different hybrids are crucial to understanding variability and genomic factors contributing to CO patterns. To assess similarities between hybrids, we initially identified overlapping COs and found a significantly higher fraction than expected by chance for every pair of hybrids (**Fig 1C**; Fisher’s exact test; all P < 2.56 x 10^−9^). The highest overlap of COs is observed between hybrids with wild parents that are evolutionarily closely related to each other (NE and CH, or HB and PN). In contrast, CO sites in PM have more overlap with PN than with other closely related species, which does not align with their evolutionary distance (**Fig 1D**). A low but significant overlap is also observed when comparing recombination hotspots in natural populations of wild and domesticated rice, cocoa and tomato [15,30,31]. COs per hybrid cover around 2% of the genome, whereas they cover 10% (77.6 Mbp) when combined. This apparently extensive non-overlapping coverage suggests divergent CO regions between the hybrids, or the need to generate more CO data per hybrid to exhaust all possible sites.

Given the low rate of CO region overlap between hybrids, we investigated whether the overall recombination landscapes across the genome are significantly correlated. **Fig 1E** shows that NE and CH have the most similar landscape. The low CO overlap (4%; **Fig 1C**) between CH and HB does not translate to a low landscape correlation (ρ = 0.64). Similarly, despite the high overlap between PM and PN COs (7%), the correlation between their landscapes is one of the lowest (ρ = 0.52), consistent with their evolutionary distance. Hybrids with wild parents that are closer to each other show higher correlation (e.g CH and NE, HB and PN) while PM, which is distant to the other wild parents, is least correlated with the other landscapes. Although the number of overlapping COs is higher than expected by chance, it is far less than the number of non-overlapping COs, which contribute more to shaping the overall recombination landscape. These results suggest that variation in CO landscapes is related to the evolutionary distance between parental genomes.

The patterns of genomic regions without recombination in the hybrids differ as well. To analyze these patterns, we identified CO coldspots of more than 1Mb, covering 72–79% of the genomes, with the highest coverage in HB and PN (**S3 Table**). All coldspots overlap SNP markers, confirming that the absence of COs is not due to the lack of markers in these regions. Grouping by genomic position and size, we assigned coldspots into 325 *unique* and 101 *shared* clusters (**Fig 1F**). Approximately 63.6% of the genome (6.4Mb euchromatic; 485Mb heterochromatic) lacks CO in all five hybrids, which we refer to here as *conserved* coldspots. PM has significantly shorter coldspots than the other hybrids (pairwise Wilcoxon rank-sum test; P < 1.4 x 10^−2^) and a large number of unique coldspot regions. Our comparison of CO landscapes indicates that although the chromosome-wide distribution is similar, a closer inspection of CO locations between hybrids reveals distinct differences: patches of CO-suppressed regions. These divergent patterns of CO regions and coldspots imply that hybridization of tomato with different wild parents results in variable recombination along the genome, revealing potential complexity in introgressive breeding.

### Absence of COs in structural variants

**Fig 2:**
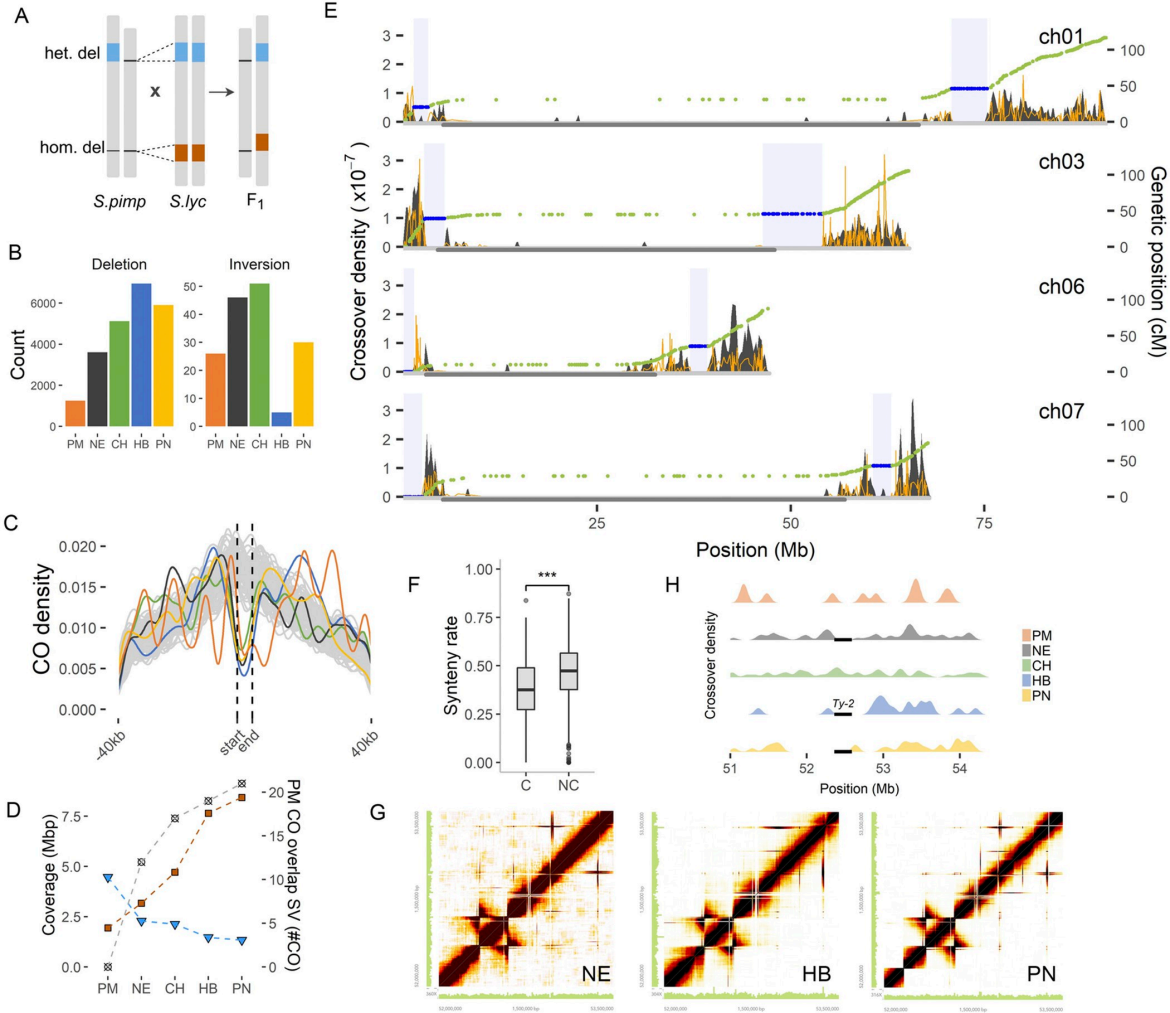
Lack of crossover in structural variations. A) Selection of parental SVs causing heterozygosity in the F1 pollen genomes. B) Frequency of SVs per wild relative. Inversions only include events > 30 kb. C) Distance of COs to the nearest SV compared to the 10,000 permutation sets represented by gray lines. The vertical lines marks the boundaries of COs. D) Genome coverage of COs (blue) and SVs (orange) in the PER (left y-axis). The gray squares show the number of PM COs that overlap with SV regions in the wild genome (right y-axis). E) Crossover density of selected PN chromosomes (gray peaks) plotted together with Marey map (green dots) of EXPEN2012. The blue dots are genetic markers within coldspot regions (blue box). The yellow distribution line indicates the recombination rate obtained by taking the derivative of the Marey map. The gray horizontal segment in the middle of the chromosome marks the PER. F) Rate of synteny in coldspot (C) and non-coldspot (NC) regions of PN (Wilcoxon rank-sum test; P < 2x10^-16^). G) Heatmap of linked reads within the *Ty-2* region in chromosome 11 long arm. H) Recombination coldspot overlapping the *Ty-2* inversion.

With the results above indicating clear variation in the occurrence of COs in the different hybrids, we speculated that large genomic rearrangements between species may underlie the varying patterns of recombination. To investigate this, we detected SVs between the parental species *S*. *lycopersicum* and the wild relatives. Given that heterozygous SVs may exist in the wild species genomes, allowing the F1 hybrid to inherit an allele that is similar to the reference genome, we genotyped SVs in the F1 hybrid pollen sequences and retained only the heterozygous ones (**Fig 2A**). Combining all parental wild species genomes, we detected 59,265 SVs larger than 50bp. Among the wild species, *S*. *habrochaites* and *S*. *pennellii* have the highest number of SVs, which are also significantly longer than in the other parental genomes (**Figs 2B** and **S3**). In order to validate the accuracy of the filtered set of structural variants (SVs), we randomly selected SVs from *S*. *pennellii* and visually compared the assemblies *of S*. *lycopersicum* and *S*. *pennellii* using dot plots (**S4 Fig**). These confirmed the presence of SVs. For instance, we found that 88% of 50 randomly selected deletions were supported, with an additional 10% belonging to more complex translocation events, and only 2% (1 case) identified as false positives. Similarly, for inversions, we observed a 76.7% true positive rate. Overall, condordant with previous studies [32–34], we find a varied landscape of SVs that are either unique to one, or shared by few, species.

To examine the relationship between SVs and recombination, we identified rearrangements and syntenic regions between *S*. *lycopersicum* and *S*. *pennellii* assemblies and compared them against PN COs. We found that 94% of PN COs are in syntenic segments in distal chromosomal regions (Fisher’s exact test; P <0.001; **S5 Fig**), corresponding to the essential role of synteny in synapsis and crossing-over of homeologous chromosomes during meiosis [10,35]. 62–74% of SVs in the genomes of wild relatives overlap with coldspots. Using a permutation test, we found strong reduction of recombination in SVs across all hybrids, specifically for SVs larger than 1kb (**S6 Fig**); further analyses will only consider SVs larger than 1kb. Most SVs are located a few to tens of kilobases away from COs (**Fig 2C**) and SV size is not correlated to distance from the CO site (**S7 Fig**). Comparing the distal euchromatin (DEU) and PER compartments of the chromosomes, we found more SVs in DEU than in PER regions, with an average ratio of 1.55 to 1. This agrees with previous observations that wild and domesticated tomato accessions have higher SV density in DEU than in PER [34,36]. In addition, SVs in PER are on average longer than those in DEU (Wilcoxon rank-sum test; P < 5.8 x 10^−16^; **S8 Fig**). A higher genome coverage by SVs regions in PER coincides with fewer CO events (**Fig 2D**). PM has the largest total number of CO events in PER, while PN has the lowest number. These PM COs overlap with SVs in the other wild genomes, suggesting that the higher SV content in other wild genomes leaves less room for recombination. Overall, our results implicate SVs as one of the major modifiers of CO landscapes in hybrids, especially in CO-rich distal regions.

We identified prominent spots in the PN DEU without CO. To validate whether these represent real coldspots, we compared them against the recombination coldspots in the EXPEN2012 linkage map [37], which is derived from a cross between *S*. *lycopersicum* and *S*. *pennellii*. Large coldspots are observed in the genetic map (**Fig 2E**), spanning 0.14 to 7.64 Mb, matching the coldspots we found in PN. Closer inspection of these large PN coldspots revealed significantly lower levels of synteny compared to non-coldspots (**Fig 2F**). These coldspots, however, may be specific to PN or may not fully overlap coldspots in other hybrids, as we found 518 COs in the same region in other hybrids. Among the PN coldspots, we found that at least two, specifically in the short arm of chromosomes 6 and 7, contain large inversions relative to the reference genome as previously validated using BAC-FISH [9]. We were able to confirm a large inversion in chromosome 7 by comparing genome assemblies and inspecting linked reads (**S9 Fig**). In addition to the inversion, this 2.4 Mbp coldspot region apparently also contains other rearrangements, like translocations, that could inhibit proper synapsis and recombination. Interestingly, this suppression in the short arm is not present in all hybrids, suggesting the absence of linkage drag when tomato is crossed with specific wild relatives. Another known SV we examined was the *Ty-2* inversion [17] in the chromosome 11 euchromatic long arm. We confirmed that a CO coldspot is located in the inversion but only present in three wild parents (**Fig 2G and 2H**). This presents the possibility of using alternative parental genomes without SVs in target regions to overcome CO suppression.

### Widespread coldspots in TE regions

**Fig 3:**
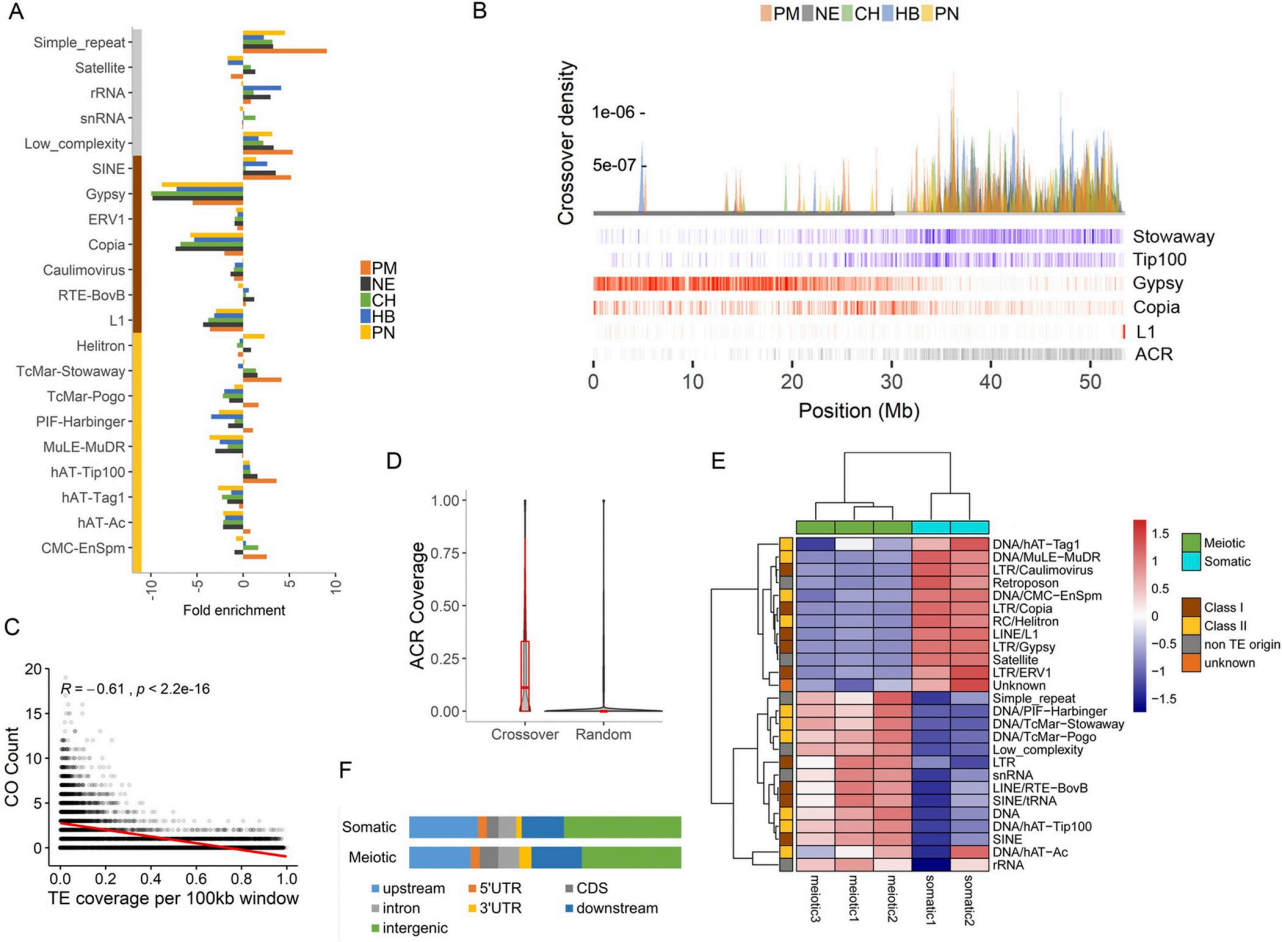
TE-associated crossovers. A) TE superfamilies and repeats showing enrichment of COs. Elements are clustered into DNA transposons (yellow), retrotransposons (brown) and other repeats (gray). B) Recombination landscape of acrocentric chromosome 2 from multiple hybrids (colored peaks) with layers of density heatmaps representing different features, including class I (red) and II (blue) TEs, and meiotic ACRs (gray). The horizontal grey line represents the PER. C) Spearman’s rank correlation of crossover count and retrotransposons (Gypsy, Copia, L1) coverage in a sliding genome window. Each dot indicates a window. The red line is the local regression fitting. D) Total coverage of ACR per region. E) Normalized enrichment of ATAC-seq read coverage over repetitive elements of meiotic and somatic cells. F) Total ACR coverage per genome feature. Upstream and downstream covers 1 kb from the transcription start and termination sites, respectively.

Aside from SVs, studies on other species have also linked the presence of transposable elements (TEs) with CO incidence, specifically retrotransposons with COs suppression [4]. Our data shows that most retrotransposons (Class I), except SINEs and RTE-BovBs, indeed exhibit suppression of COs (**Fig 3A**). However, *Stowaway* and *Tip100* (Class II) TEs, as well as simple repeats and low complexity regions, are enriched with COs. TEs associated with CO suppression (*Gypsy*, *Copia*) are densely distributed in the PER, whereas *Stowaway* and *Tip100* are located mostly in the DEU (**Fig 3B**), consistent with the CO distribution along the chromosomes. This association with TE superfamilies was also reported for historical recombination hotspots of wild and domesticated populations of tomato [15]. As shown in **Fig 3C**, the density of retrotransposons such as *Gypsy*, *Copia* and *L1* in a genomic region correlates with CO suppression. In contrast, *Stowaway* and *Tip100* show positive correlation with CO incidence (**S10 Fig**). About 98.6% of the conserved coldspots are in PER, where retrotransposon presence is dense. Furthermore, the retrotransposon superfamilies that are linked with CO suppression cover 450Mb (~52%) of the tomato genome, implying a wide span of suppression due to retrotransposons. This underscores the importance of transposable elements in shaping recombination patterns, both in hybrids and inbreeding materials and predominantly in regions with high retrotransposon density. Based on this varying association of TE superfamilies with COs, we propose that TE dynamics during tomato evolution may have a more complex impact on recombination landscapes than just suppressing COs in pericentromeric regions.

The occurrence of COs correlates with lower nucleosome occupancy and reduced DNA methylation [38]. To investigate the chromatin state of TE elements with and without COs, we performed an ATAC-seq analysis of *S*. *lycopersicum* meiotic and somatic cells and found 52,802 and 25,101 accessible chromatin regions (ACRs), respectively (**S4 Table**). These ACRs, with an average size of 733bp, represent accessible chromatin in the *S*. *lycopersicum* parent. Read distributions over the genome were highly correlated between biological replicates (**S11 Fig**). We found significant overlap between COs and meiotic ACRs (permutation test, z-score = 87.2), confirming that COs occur in regions accessible to recombination machinery. **Fig 3D** shows that crossover regions are more accessible than random genomic regions. Upon comparing meiocyte ACRs with TEs, we found that TE superfamilies enriched with COs are found in accessible chromatin segments, whereas retrotransposons like *Gypsy*, *Copia* and *L1* are not associated with accessible chromatin (**Fig 3E**). This is similar to reports in *A*. *thaliana*, where DNA transposons show nucleosome depletion and high SPO11-1-oligo levels, and retroelements like *Gypsy*, *Copia* and *L1* have very few SPO11-1-oligos with high DNA methylation and nucleosome occupancy [38]. Furthermore, shown in **Fig 3E,** the chromatin accessibility of TE superfamilies flips between somatic and meiotic cells, hinting at a preference to keep specific superfamilies inaccessible during meiossis. Our results emphasize the major role of chromatin structure in the suppression or enrichment of COs in TEs and the need to particularly analyze meiocytes, to account for tissue-specific ACRs.

Similar to the association of COs with proximal promoter regions [24], it was previously reported that ACRs are strongly associated with transcription start sites (TSSs) [39]. To evaluate this, we examined the average ATAC-seq signal in genes and their flanking regions, finding the highest coverage at the TSS in both meiotic and somatic cells (**S12 Fig**). The majority of ACRs are located near or within genes (**Fig 3F**; Fisher’s exact test; P < 0.05), similar to COs (**Fig 1A**). Normalized by the total genome coverage of the feature, promoter regions and UTRs (untranslated regions) have the highest ACR density, which may explain the excess of CO in these regions and the need for accessible chromatin to initiate recombination.

### Coldspot genes and breeding bottlenecks

**Fig 4:**
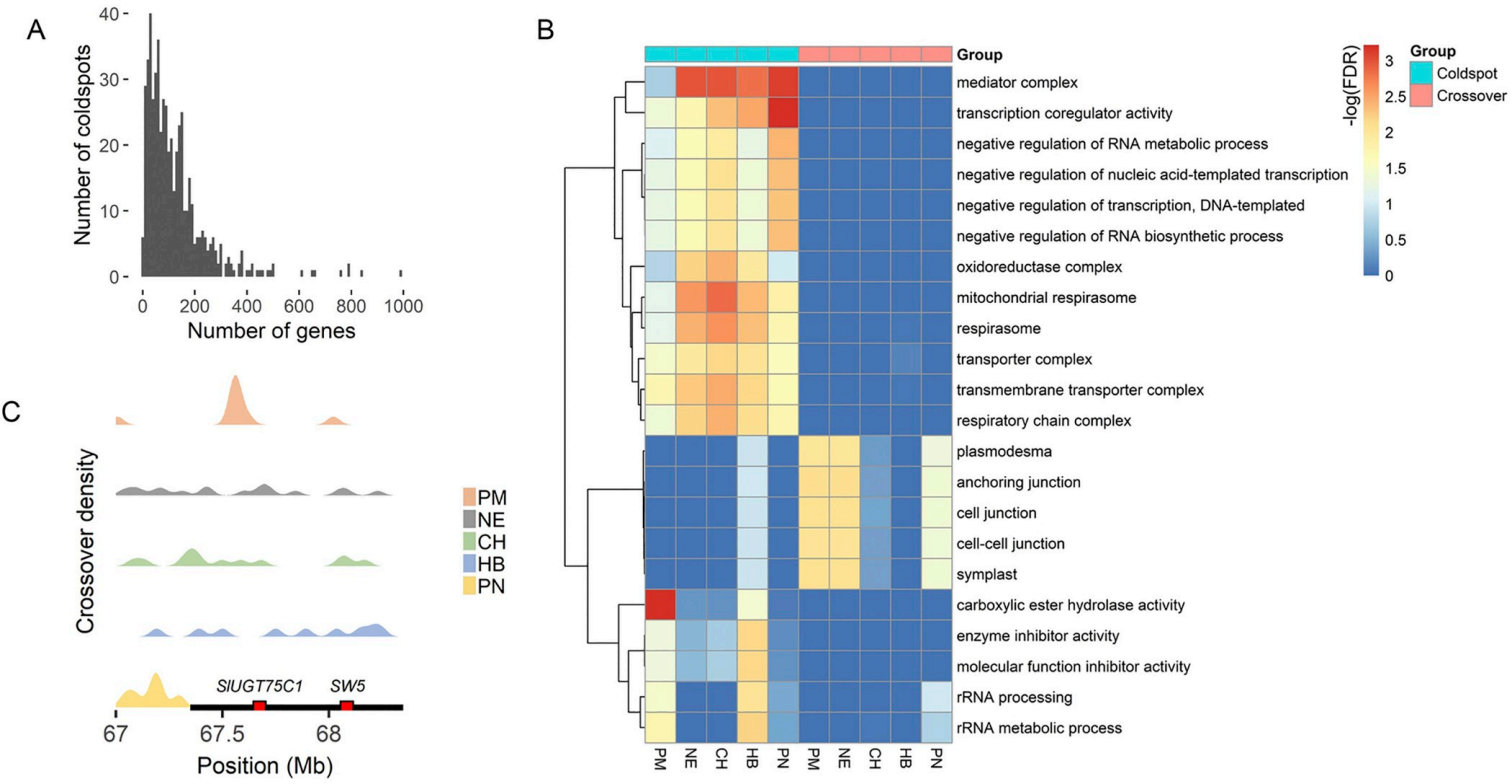
Breeding barriers and bridges. A) Number of genes per CO coldspot. B). Gene ontology (GO) terms enriched (at least 2x) in coldspot and crossover regions. C) Recombination landscape showing linkage between genes (red box) associated with agronomic and resistance traits. The black horizontal line represents the coldspot in PN.

Crossover suppression in a genomic region results to co segregation of alleles in offsprings. In total, in the CO coldspots described above, 21,157 genes are found (63% of all genes); 471 of these have a known link with resistance and agronomic traits. Here we refer to a group of genes within the same CO-suppressed region as coldspot gene groups. 484 of the coldspots contain coldspot gene groups with at least 20 genes (**Fig 4A**). Although many coldspot gene groups are located in the conserved coldspots in PER, there are also coldspot gene groups located in 81 coldspots in gene-dense DEU. In a gene ontology (GO) enrichment analysis of coldspot regions (**Figs 4B and S13**), we found terms associated with basal housekeeping functions (e.g like transcription coregulator activity, transporter complex, rRNA processing, metabolic processes) but also multiple metabolic processes). The coldspots in the short arms of chromosome 6 and 7, coinciding with known inversions [9], contain 130 and 295 genes, respectively, and are associated with responses to oxidative stress (P = 1.27 x 10^−4^) and specific catabolic and metabolic processes (9.66 x 10^−8^). The latter may reflect the evolutionary divergence between tomato and the wild species and the modification in metabolism during domestication [40–43]. As previously reported in other crop species, some metabolic traits selected for during domestication originate from structural rearrangements [44]. Supergenes within these rearrangements have indeed been linked to metabolic pathways and alternative phenotypes in plants [11,41]. These results reveal that the absence of COs in specific genomic regions may affect plant traits and even evolution. This may also lead to bottlenecks in breeding, when hundreds of alleles are kept in fixed combinations.

Aside from the rewiring of the metabolome, we were interested whether domestication is associated with linkage drag in regions containing resistance (*R*) genes. Upon inspecting the coldspot gene groups in PN, we found that they include 2,736 genes, 877 of which have been identified as domestication syndrome genes [45], and they are enriched with *R* genes (Fisher’s exact test; P = 5.1 x 10^−4^) (**S14 Fig**). For example, the coldspot in chromosome 7 contains 295 genes, including *R* genes and *chitinase* genes. In this region, we found an enrichment of genes related to the *chitin catabolic process* (FDR = 1.49 x 10^−4^), *chitin binding* (FDR = 1.63 x 10^−4^) and *chitinase activity* (FDR = 2.18 x 10^−2^), which are involved in plant defense responses against pathogens [46–48]. Our findings are consistent with observations in Arabidopsis, in which CO coldspots with many SVs contain clusters of *R* genes [49]. Aside from *R* genes, the coldspot in chromosome 7 also contain the *SUN* locus, which is linked with variable fruit shape in the wild and cultivated tomato [50,51]. The remaining coldspots in PN contain 17 genes with putative roles in fruit shape determination and at least 116 genes linked with agronomic traits, further substantiating the association between coldspots and domestication syndrome traits [52]. Coupled with the list of important genes, our catalogue of coldspots will allow breeders to pre-examine whether an introgression may result in linkage between agronomic traits and undesirable traits like susceptibility to diseases.

CO-suppressed regions can limit breeding by linking agronomically beneficial alleles with deleterious alleles. We identified an example of undesirable linkage (**S15A Fig**) by examining genes located in coldspots. Shown in **Fig 4C**, a coldspot in chromosome 9 of PN spans genes associated with resistance and fruit ripening, linking *SlUGT75C1* and *SW5*. The significantly lower expression of *SlUGT75C1* in *S*. *lycopersicum* relative to *S*. *pennellii* may yield accelerated fruit ripening [53,54]. On the other hand, *S*. *lycopersicum* has the marker (Sw5a^S^) linked with tomato spotted wilt virus (TSWV) susceptibility [55] and lacks the resistance allele (missense mutation) to Leaf Curl New Delhi virus (ToLCNDV) [56], which we observed in *S*. *pennellii*. The fact that these alleles occur in a CO coldspot suggests that in PN, we cannot generate recombinant F1 gametes with both *SlUGT75C1* and Sw5a^r^ genes. The tight linkage may possibly be resolved by pairing *S*. *lycopersicum* with the other wild relatives, which exhibit recombination between these genes and may introgress the same resistance allele from the other four wild parental species (**Fig 4C**). This example suggests how recombination data analysis can support resolving linkage drag through the strategic selection of parental accessions or, alternatively, maintaining coldspots to preserve linkage between alleles conferring a desired trait combination.

A remarkable case of introgression of disease resistance and associated linkage drag of three quarters of a wild chromosome is the Tomato Mosaic Virus (ToMV) resistance from the *S*. *peruvianum* accession PI 18650, providing resistance to more than 90% of the commercial tomato varieties [57,58]. The resistance from this wild species is conferred by the *Tm-2*^*2*^ gene located on ch09 of tomato [57]. This introgression region of around 60Mbp exhibits low levels of COs in all evaluated hybrids. However, we do find some COs in this region (**S15B Fig**), implying the possibility of breaking the linkage. A recently released assembly of a chromosome with this introgression revealed small and medium sized inversions, and a series of relatively small translocations and duplications [59]. Although there are many SVs in this introgression, there are still sufficient syntenic regions that could in principle allow COs. However, due to the low frequency of CO in these regions, they may not be observed in limited size offspring populations previously used in tomato breeding. Utilizing our pollen-sequencing data, we precisely locate recombination sites in regions previously considered coldspots, offering a promising avenue to disrupt linkage.

## Discussion

We have applied our pooled pollen sequencing strategy for high-throughput, low-cost mapping of the recombination landscape in five tomato hybrids. The accurate alignment of the recombination landscape we find in a cross between *S*. *lycopersicum* and its wild relative *S*. *pennellii* (PN) with a genetic linkage map demonstrates the reliability and precision of our approach. Our study represents the most comprehensive profile of recombination in tomato hybrids thus far, contributing valuable insights into one of the few plant species with comprehensive crossover (CO) data from multiple hybrid populations. We observed that COs are predominantly distributed in the gene-rich DEU regions of each chromosome, consistent with previous reports [19,24]. Despite overall similarity in the CO landscape between hybrids, we uncovered fine-scale differences in CO patterns and regions without recombination. CO coldspots, characterized by their limited ability to reshuffle alleles between tomato and wild species, pose challenges to introgressive hybridization breeding: they hamper the efficient incorporation of desirable traits from wild species into cultivated tomato varieties and decrease the overall efficiency of backcrossing processes. While the majority of coldspots are conserved across all hybrids, some coldspots are unique to a specific cross. These may serve as putative targets to overcome linkage drag or to investigate the underlying fitness advantage driving suppression of recombination. Future work should include efforts to characterize and compare unique and shared coldspots.

Across all hybrids, COs were found absent in regions with structural variation (SV), particularly in lineage-specific rearrangements. The diverse recombination patterns among hybrids are linked to rearrangements between the wild parental genomes, suggesting that SV profiles in F1 progeny may serve as indicators for regions allowing or inhibiting crossovers. This insight empowers breeders to refine introgression plans, by examining recombination patterns in specific loci of interest before undertaking complex hybridization and screening processes. Despite multiple studies reporting the negative association between SVs and COs, the mechanisms through which SVs inhibit recombination remain unclear. Rowan, *et al*. [6] proposed several possible explanations for the observed suppression of COs in heterozygous SVs, including the absence of a repair template, the tendency to produce non-viable gametes, DNA methylation in the SV region, and the blocking of physical interaction in variant regions that prevents proper synapsis. Additionally, it has been reported that DSBs in inversion regions are preferentially resolved as noncrossover gene conversions and not as COs [6,60,61].

Despite the observed extensive impact of SVs on CO patterning, we yet have limited information on the more complex rearrangements between the parental genomes. It is important to note that SV detection, especially large and complex inversions and translocations, is challenging and requires long reads or genome assemblies. A more complete profile of structural rearrangements will show the effect of SVs and retrotransposons on CO distribution in finer detail. In this study, we used *S*. *lycopersicum* as a control parent and a reference genome to detect how SVs in wild genomes shape the CO landscape. However, the accuracy of CO detection and comparison is obscured if the reference genome would not be one of the parents and both parents are structurally divergent to the reference genome.

Structurally heterozygous regions in the genome, causing a lack of recombinant haplotypes, have been associated with adaptive phenotypes and plant domestication and speciation [11,12,15,44]. Inversions, capturing two or more alleles adapted to an environment, prevent recombination and confers a selective advantage, which promote their spread in the population [62]. SVs in gene-rich distal regions may be attributed as a major modifier of recombination distributions between hybrids, especially between PM and other hybrids. Although PER regions have larger SVs than DEU, they harbor less COs, thus minimally changing the landscape. DEU SVs, such as the Ty-2 inversion (**Fig 2G**), impact the landscape more while harboring species-specific alleles conferring divergent phenotypes.

In this study, we demonstrated that certain CO coldspots overlap *R* gene hotspots. These hotspots not only accumulated nucleotide variations during the evolution of wild tomato relatives, but also underwent copy number expansion and contraction, resulting in varying resistance to pathogens [63]. Some *R* gene hotspots can become CO hotspots, facilitating rapid diversification to overcome new pathogens. In contrast, *R* genes conferring resistance to pathogens with low genetic plasticity are located in CO coldspots, possibly maintained by structural heterozygosity [64,65]. The association between resistance genes and some unfavorable alleles due to genetic linkage limits the introgression of resistance haplotypes into breeding lines. A specific case of linkage drag involving the resistance to *Fusarium* wilt race 3, reduced fruit size and increased sensitivity to bacterial spot, was broken by reducing the size of the introgression [66]. However, this shrinking of the introgressed region is feasible only because it is not induced by an inversion or other CO-suppressing type of SV.

Some coldspots contain genes associated with metabolic processes and fruit traits, suggesting linkage between genes that may be related to the significant changes in chemical composition of tomato fruit due to fruit mass-targeted selection during domestication [43]. These coldspots represent potential targets for metabolite engineering in *de novo* domestication of wild tomato relatives. The enrichment of genes linked with resistance and metabolism in CO coldspots is partly a result of plant evolutionary events involving SVs [41,44]. Further examination of recombination coldspots can provide breeders with insights into the genetic or epigenetic causes of CO suppression and the divergent phenotypes resulting from the evolution of locally adapted alleles and from domestication.

Alongside the association between SVs and CO coldspots, we identified specific superfamilies of TEs strongly linked with crossovers and accessible chromatin regions (ACRs). Our analysis of ACRs in meiocytes revealed that the varying association between superfamilies may be influenced by their chromatin configuration, keeping elements like *Gypsy* and *Copia* inaccessible during meiosis, thereby preventing COs. We also observed differential chromatin accessibility of TE elements in somatic and meiotic cells, prompting the need for further studies to elucidate whether this relates with different functions or the regulation to limit proliferation of specific TEs during meiosis [67]. Despite establishing an association between TEs and COs, it remains unclear whether TEs directly shape the recombination landscape, or if recombination and TE insertions simply coincide in ACRs and genic regions due to TE insertion bias [38,68]. Notably, in tomato, *Stowaway* elements tend to insert within or near genes, while *Gypsy* elements prefer pericentromeric regions [69,70]. On the other hand, the consistent chromatin state per TE superfamily may indicate that, depending on the type, new TE insertions can either suppress or promote recombination [38,68]. For instance, the expansion of pericentromeric regions in *A*. *alpina* due to retrotransposon insertions resulted in more regions with suppressed recombination [71]. Further investigation how the activity of TEs, particularly during stress exposure, influences the recombination landscape [72,73] would provide valuable insight.

While prior studies aimed to increase CO frequencies, they encountered challenges in achieving homogeneity across the genome [21,74,75], missing CO coldspots. Notably, recent advances demonstrated the potential to restore recombination by inverting an inversion through genome editing [76]. However, the application of such a solution in direct breeding practices may face constraints due to current regulations. As an alternative, we propose the identification of alternative parents capable of resolving the deficiency in recombination within regions containing SVs while preserving the desired genetic background. We emphasize the importance and advantage of conducting compatibility or linkage drag checks as a cost-effective component of a breeding scheme. Subsequent research efforts could focus on the development of predictive models to map CO coldspots between pairs of accessions without the need for creating a mapping population.

Linkage drag represents a significant challenge in introgressive breeding and can impede the development of commercial plants. A map distinguishing recombining and non-recombining regions offers a rapid means of assessing recombination suppression that may lead to linkage drag. Furthermore, pollen of different hybrids can be sequenced as well, screening different parental combinations for CO frequencies neighboring the desired allele from the wild relative, and thereby checking their suitability for removing or minimizing linkage drag. Thus, the methodology and data presented here not only contributes to scientific understanding of recombination landscapes, CO hotspots, and coldspots but also holds practical value in the field of plant.

## Methods

### Sequencing of pollen gametes

We produced F1 plants from crosses between *S*. *lycopersicum* cv. Heinz1706 and the following wild relatives: *S*. *pimpinellifolium* (CGN14498), *S*. *neorickii* (LA0735), *S*. *chmielewskii* (LA2663), *S*.*habrochaites* (LYC4), and *S*. *pennelli* (LA0716). The wild species served as the male parents. Mature pollen were collected from each hybrid and processed to isolate the high molecular weight DNA using the protocol in Fuentes, *et al*. [24]. 10X Genomics libraries were constructed according to the Chromium Genome v2 Protocol (CG00043) and then sequenced on an Illumina HiSeq 2500. Aside from the sequencing pool of pollen from these hybrids, we used the same protocols to sequence the inbreds of the parental tomato and the wild species.

### Crossover detection

For detecting segregating markers in the hybrids, linked reads from the inbred wild parents were aligned against the *S*. *lycopersicum* cv. Heinz reference genome SL4.0 [77] using *Longranger* [78] and were subsequently processed using GATK HaplotypeCaller [79] with the recommended hard filtering to screen single nucleotide polymorphisms (SNPs). Heterozygous SNPs and other SNPs located in homopolymeric regions and regions prone to false positives due to inaccurate assembly or copy number variations, resulting in highly heterozygous alignments, were filtered out. Thereafter, for each hybrid, the linked reads from pollen gametes were aligned against SL4.0 using *Longranger* and were phased using the segregating markers as described in Fuentes, *et al*. [24]. For each putative recombinant molecule, we applied filters on the resolution, block size, and the number of supporting reads, wild cards and markers per phased block. By filtering based on resolution, which we defined as the inverse of the distance between the SNP markers bounding the CO site (resolution = 1/distance), we recovered high resolution CO (distance below 1kb or resolution above 0.001) for comparison with genomic features. To address the possibility of false positives due to the presence of multiple molecules per GEM in 10x linked reads, we limit the spanning distance based on the expected sizes of DNA molecules. In the updated version of our pipeline [24], filtering putative recombinant molecules with significant overlap with repeats and transposable elements was deprecated to enable analysis of correlation between COs and superfamilies of TEs. The number of overlapping crossover events between hybrids and their significance were determined using *bedtools* [80]. We constructed recombination landscape by estimating the CO density using the R density() function, with a binwidth of 100kb. To compare landscapes, Spearman’s rank correlation matrix was computed on vectors of CO counts in 500-kb sliding windows with a 50-kb step size.

### Detection of coldspots

We counted the number of COs per hybrid in 10kb sliding windows with 5kb step size and merged those windows with at least one CO and within 1kb distance of each other. The resulting set of genomic intervals are considered CO regions. Regions without COs spanning at least 1Mb are considered coldspots. To cluster coldspots from all hybrids, we first grouped those with at least 1 bp overlap. For each group, we built a graph with coldspots as nodes, connected by edges if they have a least 50% reciprocal overlap. Each graph was split into connected components (C) and then based on the genomic position, we computed the distance (*p*_k_) between the leftmost and rightmost coldspot in each component. If *p*_k_ is at least 1.5 times the size of the smallest coldspot in C_k_, the component was further regrouped by hierarchical clustering using a distance matrix d(i,j) = (*f*-2*length(i∩j))/(*f*-length(i∩j)), where i and j is the pair of coldspots in a component and *f* is the sum of their lengths. Complete linkage was used; the resulting dendrogram was cut at the height of 0.3. The resulting groups were used to define shared coldspots, which occur in at least two hybrids, and unique coldspots, *i*.*e*. those coldspots that occur only in one hybrid. We also identified conserved coldspots or regions without CO in all five hybrids. For coldspot validation, we used existing genetic maps. We identified large DEU coldspot regions in the linkage map by mapping the EXPEN2012 [37] markers against the tomato reference genome, retrieving the physical position and subsequently plotting against the genetic position. Then, we compared the EXPEN2012 coldspot against the PN coldspots.

### Detection and validation of SVs

Linked reads from inbreds of all the parental species were aligned to the reference genome and analyzed to detect SVs using *Longranger*. With the presence of heterozygous SVs in the parental genomes, it is possible that only the reference allele may have been inherited by the F1 plants. To determine for each hybrid whether the F1 plants inherited an SV allele causing heterozygosity between the homologous chromosomes during meiosis, we profiled SVs in the F1 pollen linked-reads. The pool of pollen included both recombinant and non-recombinant regions and represented alleles from both parental genomes of each F1 plant. Thereafter, SVs were reported if present in both the inbred and the corresponding pollen data, referred to as *parental SVs*. To further remove problematic regions, SVs between the Heinz reference and the Heinz inbred, which we refer to as *self SVs*, were detected. Lastly, we reported parental SVs, of the deletion (DEL) and inversion (INV) type, that do not overlap self SV. For SV validation, we compared the SL4.0 assembly against the existing assembly of *S*. *pennellii* [81] using Syri [82] and manually inspected randomly selected sets of DELs and INVs using Gepard [83].

### Enrichment analysis

To determine the enrichment of COs in specific TE superfamilies, we generated 10,000 permutations of the CO data per hybrid using bedtools and computed the number of overlaps with transposable elements. We then compared the observed and the expected overlap with TE of these CO events. For detecting overrepresented motifs, we retrieved the genomic sequences spanning CO sites with a resolution above 0.002, including the 3-kb flanking regions, and analyzed these with the MEME suite [84] using default parameters. Furthermore, we generated a list of genes present in the CO and coldspot regions and subsequently ran Panther [85] to identify enriched GO Terms. We also computed the number of resistance genes [64] and historical recombination hotspots [15] in the CO coldspots. We previously identified historical hotspots based on the estimated population-scaled recombination of domesticated and wild tomato accessions [15].

### ACR detection

Tomato plants were grown and cultivated in a greenhouse with a photoperiod of 16 hours light and 8 hours dark, and a minimum temperature of 16°C. Only healthy four- to seven-week-old plants were used in all experiments. The youngest leaves (the most apical) were used to isolate somatic nuclei. Meiocytes were isolated from young flower buds containing anthers that were less than 2 mm in size. Microscopic analysis revealed that at this stage in anther development nearly all meiocytes are in prophase I.

For nuclei isolation, approximately 0.4 g of young tomato leaves, or anthers from 20 prophase I flower buds were collected and immediately chopped in 2mL pre-chilled lysis buffer (15mM Tris-HCl pH7.5, 20mM NaCl, 80mM KCl, 0.5mM spermine, 5mM 2-mercaptoethanol, 0.2% Triton X-100) until a homogenous suspension was obtained. The suspensions were filtered twice through Miracloth and subsequently loaded gently on the surface of 2mL dense sucrose buffer (20mM Tris-HCl pH 8.0, 2mM MgCl2, 2mM EDTA, 25mM 2-Mercaptoethanol, 1.7M sucrose, 0.2% Triton X-100) in a 15mL Falcon tube. The nuclei were centrifuged at 2200g at 4°C for 20 minutes and the pellets were resuspended in 500μL pre-chilled lysis buffer.

Nuclei were kept on ice during the entire sorting procedure. Nuclei were first stained with 4,6-Diamidino-2-phenylindole (DAPI) and examined for integrity and purity using a Zeiss Axioskop2 microscope. Once the integrity and purity of nuclei was confirmed, nuclei were sorted in a BD FACS Aria III sorter. A total of 50,000 nuclei were sorted based on their size, shape and the intensity of the DAPI signal, which indicates the ploidy levels of the nuclei. 2n nuclei were sorted from young leaf samples, while 4n nuclei, corresponding to meiocytes, were sorted from anther samples. After sorting, nuclei were once more checked for integrity and purity under a microscope. Nuclei were transferred from sorting tubes to LoBind Eppendorf tubes and centrifuged at 1000g at 4°C for 10 min and then washed with Tris-Mg Buffer (10mM Tris-HCl pH 8.0, 5mM MgCl2).

Tn5 integration was performed as previously published [86] on purified nuclei using the Nextera Illumina kit (Illumina, FC 121 1031) at 37°C for 30 min. After tagmentation (insertion of the sequencing adapter into accessible chromatin), the tagged DNA was purified with a Qiagen MinElute PCR purification kit. To generate an ATAC-seq library for sequencing, tagged fragments were amplified by two successive rounds of PCR. In the first round of PCR, the fragments were amplified by only 3 PCR cycles using the NEBNext High-Fidelity 2xPCR Master Mix and the Custom Nextera PCR Primer 1 and barcoded sets of Primer 2. Subsequently, 2.5 μL of the PCR amplified DNA was subjected to quantitative PCR to estimate the relative amount of successfully tagged DNA fragments and to determine the optimal number of amplification cycles for the second round of PCR. The latter was estimated by plotting fluorescence values against the number of cycles. The number of cycles required for the second PCR amplification equals the number of cycles that results in 25% of the maximum fluorescent intensity [87]. ATAC-seq libraries generated were purified using AMPure XP beads (Beckman Coulter) and quantified using Qubit DNA high sensitivity assay in combination with Tapestation D1000 prior to sequencing.

Sequencing was carried out using an Illumina NextSeq 500. A snakemake analysis workflow (https://github.com/KoesGroup/Snakemake_ATAC_seq) was used for the analysis of the ATAC-seq dataset with the default parameters of the configuration files. Briefly, paired-end sequencing reads were trimmed to remove the Illumina adapter sequences using Trimmomatic 0.38 [88]. Only reads with a quality score (Phred) above 30 were kept and mapped to the SL4.0 version of the tomato genome, tomato chloroplast genome and tomato mitochondrial genome using Bowtie2 [89]. Only reads mapping to a unique position in the tomato genome were used for further analysis. Reads mapping to the tomato genome were then shifted to correspond to the real Tn5 binding location using the Deeptools alignmentSieve with the parameter “–ATACshift”. ATAC peaks were called using the MACS2 algorithm [90,91].

Reads mapping uniquely to the transposable element annotation were counted using bedtools. Read counts were normalized by the total number of reads in the library and then grouped by the transposable element classes. Heatmaps and clustering was performed using the pheatmap package 1.0.8 (CRAN).

## Supporting information

S1 Text Detection of crossovers. (PDF)

S1 Table Linked read information of F1 hybrids. (XLSX)

S2 Table Crossover events in all five hybrids. (XLSX)

S3 Table Crossover coldspots in all five hybrids. (XLSX)

S4 Table Accessible chromatin regions in S. lycopersicum. (XLSX)

S1 Fig Meiotic crossovers in different hybrids. (PDF)

S2 Fig Crossovers near and within genes. (PDF)

S3 Fig Longer structural variants for more distant wild genomes. (PDF)

S4 Fig Validation of structural variants. (PDF)

S5 Fig Parental genome alignment. (PDF)

S6 Fig Suppression of COs in SVs. (PDF)

S7 Fig Distance of SVs to COs by size. (PDF)

S8 Fig Sizes of structural variants. (PDF)

S9 Fig Inversion in chromosome 7 short arm. (PDF)

S10 Fig TE and CO correlation. (PDF)

S11 Fig Pearson correlation of ACRs. (PDF)

S12 Fig ATAC-seq peaks at transcription start sites (TSS). (PDF)

S13 Fig Functional enrichment in coldspot and CO regions. (PDF)

S14 Fig Resistance genes across the tomato genome. (PDF)

S15 Fig Unfavorable linkage. (PDF)

S16 Fig DNA molecule lengths generated using Fragment Analyzer. (PDF)

S17 Fig Linked-read coverage along the chromosomes. (PDF)

S18 Fig False positive hotspots in pericentromeres. (PDF)

S19 Fig Filtering criteria on recombination molecules. (PDF)

S20 Fig Overrepresented motifs. (PDF)
