# Supplementary material for: A catalogue of recombination coldspots in interspecific tomato hybrids
Source: PLoS Genet. 2024 Jul 1;20(7):e1011336. doi: 10.1371/journal.pgen.1011336 (PMC11244794; doi:10.1371/journal.pgen.1011336)
Supplement: S1 Text — (PDF) [file pgen.1011336.s001.pdf]

S1 Text. Detection of Crossovers

We have generated hybrid crosses of *S. lycopersicum Heinz1706* and its wild relatives *S. pimpinellifolium* (CGN14498; **PM**)*, S. neorickii* (LA0735; **NE**), *S. chmielewskii* (LA2663; **CH**)*, S. habrochaites* (LYC4; **HB**)*,* and *S. pennellii* (LA0716; **PN**). Hereafter, we use these abbreviations and the species name when referring to the hybrids and the parental genome, respectively. The pool of pollen from each hybrid was sequenced using 10X Genomics kits (**S1 Table**) based on the protocol described in Fuentes, et al. [1]. We show the lengths of DNA molecules we sequenced and the distribution of read along the chromosomes in **S16 Fig** and **S17 Fig**, respectively. Regions prone to false positive COs, as indicated by a high density of heterozygous SNPs and excessive sequence coverage, were filtered out (**S18 Fig**). *S. pennellii* and *S. pimpinellifolium*, the most distant and closest species to *S. lycopersicum* in this study, exhibited the highest and lowest number of SNPs with respect to the reference genome (*S. lycopersicum*; SL4.0) (**Table 1**). Using the filtered SNPs, we identified haplotype shifts, leading to the detection of putative recombinant haplotypes. The recombinant molecules were further filtered as described in the Methods (**S19 Fig**). In each recombinant molecule, CO sites are reported based on the bounding SNP markers.

We detected a total of 6,382 COs in all hybrids, primarily located in distal segments of chromosomes (**S2 Table; S1 Fig**), consistent with previous reports in tomato and other plant species [2,3]. For each hybrid, COs are confined to 6-12% of the distal euchromatin (DEU) and less than 1% of the pericentromeres (PER). In total, CO regions account for only 2% of the whole genome (relative to SL4.0), matching observations in other eukaryotic organisms where recombinations are concentrated in hotspots[2-5]. Despite known low recombination rates in tomato PER regions[6,7], we detected there a total of 710 COs (11.1%) in all hybrids. These are likely located in euchromatin islands within the PER. It has been proposed that the suppression of double-strand-breaks (DSBs), precursors of COs, by condensed repeat-rich chromatin like PER helps safeguard against genome destabilization [7,8]. Our results thus show that some COs occur in PER, unlike previously assumed.

We validated the recombination profile of PM by comparing it to existing CO data. The COs in the pollen gametes significantly overlap with COs previously detected in a RIL population of the same parental cross (Fisher's exact test; $P = 5.8 \times 10^{-18}$) [9]. More in detail, the frequency of COs in sliding 500kb genomic windows in DEU or in PER shows a moderate correlation between the pollen and the RIL data (Spearman's rank correlation; DEU, $\rho = 0.33$; $P < 2.2 \times 10^{-16}$; PER, $\rho = 0.22$; $P < 2.2 \times 10^{-16}$). This modest correlation may be attributed to insufficient data to detect all possible recombination sites. However, comparison with historical recombination hotspots detected in natural populations of wild and domesticated tomato [10] revealed that the COs in hybrids overlap with 294 (Fisher's exact test; $P = 2.0 \times 10^{-13}$) and 36 (Fisher's exact test; $P = 3.8 \times 10^{-11}$) historical hotspots in DEU and PER, respectively.

The new CO data we generated thus not only confirms previous results but also demonstrates the robustness of our method to detect rare recombination sites in PER.

The vast majority (5,150; 81%) of COs are located within genes and their 1kb flanking regions, while another 471 are positioned between 1kb and 3kb from genes (**Table 1**; **Fig 1a**; **S1 Fig**). PM and NE have the lowest CO resolution, defined as the inverse of the distance between the SNP markers bounding the CO site (resolution = 1/distance). PM COs overlap more with intergenic regions than COs in other hybrids. One possible confounding factor could be that *S. pimpinellifolium* has fewer SNPs w.r.t. the reference than the other species (**Table 1**), which may lead to a lower resolution of detected COs. To determine if this contributed to the higher overlap between intergenic regions and COs in PM, COs with similarly distributed resolution for all hybrids were separately analyzed (**S1b Fig**). However, the result still shows the same higher intergenic overlap of crossover events in PM (**S2 Fig**). This is an intriguing result that could use further exploration.

Aside from association with genes, sequence motifs are discovered at CO sites as well [9,11,12]. We found that CTT-like repeats, poly-AT and A-rich motifs are actually enriched in regions flanking rather than within high-resolution COs (**S20 Fig**). These CO motifs have been previously identified in other plant species. Their close proximity to CO sites suggests that they may play a role in recruiting recombination-promoting factors. This was previously proposed [13], but it was not possible to definitively determine then whether these motifs are actually located within or just near CO sites, due to the low resolution. This analysis underscores the importance of high-resolution CO sites to infer association to small genomic features.

References

1. Fuentes, R. *et al.* Meiotic recombination profiling of interspecific hybrid F1 tomato pollen by linked read sequencing. *Plant J* **102**, 480-492 (2020).
2. Mercier, R., Mezard, C., Jenczewski, E., Macaisne, N. & Grelon, M. The molecular biology of meiosis in plants. *Annu Rev Plant Biol* **66**, 297-327 (2015).
3. Wang, Y. & Copenhaver, G.P. Meiotic Recombination: Mixing It Up in Plants. *Annu Rev Plant Biol* **69**, 577-609 (2018).
4. Choi, K. & Henderson, I.R. Meiotic recombination hotspots - a comparative view. *Plant J* **83**, 52-61 (2015).
5. Lambing, C., Franklin, F.C.H. & Wang, C.-J.R. Understanding and Manipulating Meiotic Recombination in Plants. *Plant Physiology* **173**, 1530-1542 (2017).
6. Termolino, P., Cremona, G., Consiglio, M.F. & Conicella, C. Insights into epigenetic landscape of recombination-free regions. *Chromosoma* **125**, 301-8 (2016).
7. Yelina, N., Diaz, P., Lambing, C. & Henderson, I.R. Epigenetic control of meiotic recombination in plants. *Sci China Life Sci* **58**, 223-31 (2015).
8. Tock, A.J. & Henderson, I.R. Hotspots for Initiation of Meiotic Recombination. *Front Genet* **9**, 521 (2018).
9. Demirci, S. *et al.* Distribution, position and genomic characteristics of crossovers in tomato recombinant inbred lines derived from an interspecific cross between *Solanum lycopersicum* and *Solanum pimpinellifolium*. *Plant Journal* **89**, 554-564 (2017).
10. Fuentes, R.R., de Ridder, D., van Dijk, A.D.J. & Peters, S.A. Domestication Shapes Recombination Patterns in Tomato. *Mol Biol Evol* **39**(2022).
11. Wijnker, E. *et al.* The genomic landscape of meiotic crossovers and gene conversions in *Arabidopsis thaliana*. *Elife* **2**, e01426 (2013).
12. Shilo, S., Melamed-Bessudo, C., Dorone, Y., Barkai, N. & Levy, A.A. DNA Crossover Motifs Associated with Epigenetic Modifications Delineate Open Chromatin Regions in Arabidopsis. *Plant Cell* **27**, 2427-36 (2015).
13. Choi, K. *et al.* Nucleosomes and DNA methylation shape meiotic DSB frequency in *Arabidopsis thaliana* transposons and gene regulatory regions. *Genome Research* **28**, 532-546 (2018).
