# Supplementary material for: A catalogue of recombination coldspots in interspecific tomato hybrids
Source: PLoS Genet. 2024 Jul 1;20(7):e1011336. doi: 10.1371/journal.pgen.1011336 (PMC11244794; doi:10.1371/journal.pgen.1011336)
Supplement: S1 Fig — (PDF) [file pgen.1011336.s006.pdf]

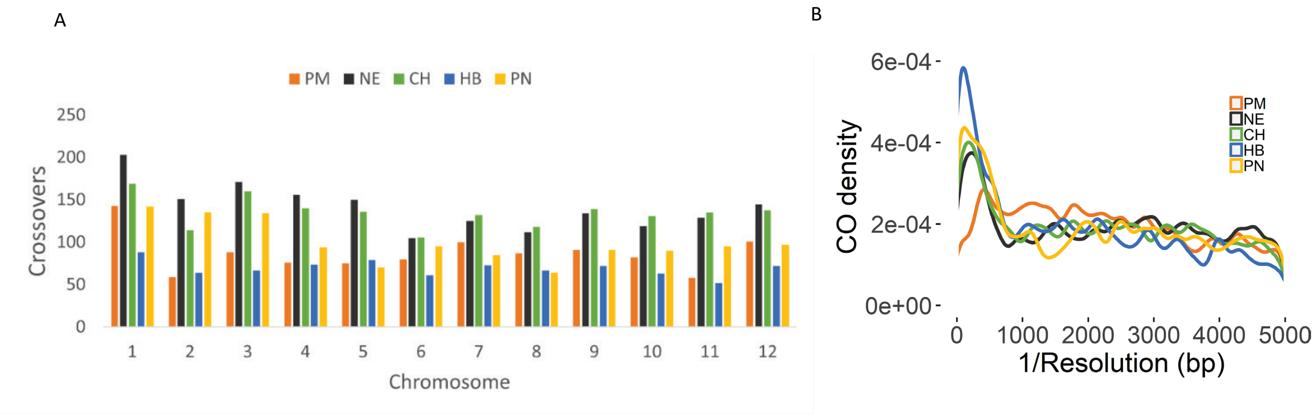

S1 Fig. **Meiotic crossovers in different hybrids. A)** Crossover count per chromosome. **B)** *S. pimpinellifolium* crossovers have lower resolution compared to the other groups, but between the resolution of 0.0002 to 0.001 (1kb to 5kb), the distributions are similar across the different crosses.
