# Supplementary material for: A catalogue of recombination coldspots in interspecific tomato hybrids
Source: PLoS Genet. 2024 Jul 1;20(7):e1011336. doi: 10.1371/journal.pgen.1011336 (PMC11244794; doi:10.1371/journal.pgen.1011336)
Supplement: S2 Fig — (PDF) [file pgen.1011336.s007.pdf]

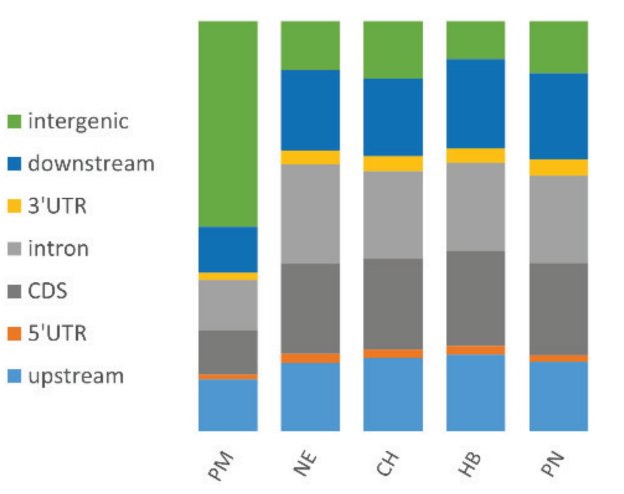

S2 Fig. **Crossovers near and within genes.** Overlap of crossovers (resolution between 0.0002 to 0.001) with gene features and intergenic regions.
