# Supplementary material for: A catalogue of recombination coldspots in interspecific tomato hybrids
Source: PLoS Genet. 2024 Jul 1;20(7):e1011336. doi: 10.1371/journal.pgen.1011336 (PMC11244794; doi:10.1371/journal.pgen.1011336)
Supplement: S3 Fig — (PDF) [file pgen.1011336.s008.pdf]

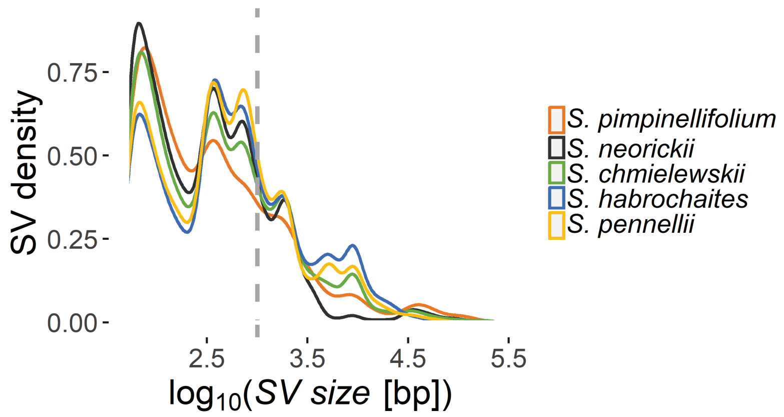

S3 Fig. **Longer structural variants for more distant wild genomes**. Distribution of SV sizes per wild parental genome (relative to *S. lycopersicum* reference genome) showing higher frequency of longer SVs for *S. habrochaites* and *S. pennellii*.
