# Supplementary material for: A catalogue of recombination coldspots in interspecific tomato hybrids
Source: PLoS Genet. 2024 Jul 1;20(7):e1011336. doi: 10.1371/journal.pgen.1011336 (PMC11244794; doi:10.1371/journal.pgen.1011336)
Supplement: S4 Fig — (PDF) [file pgen.1011336.s009.pdf]

A

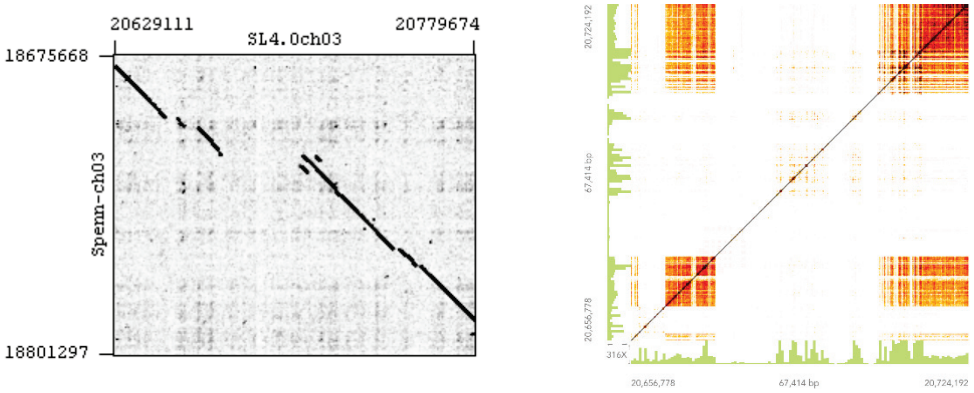

B

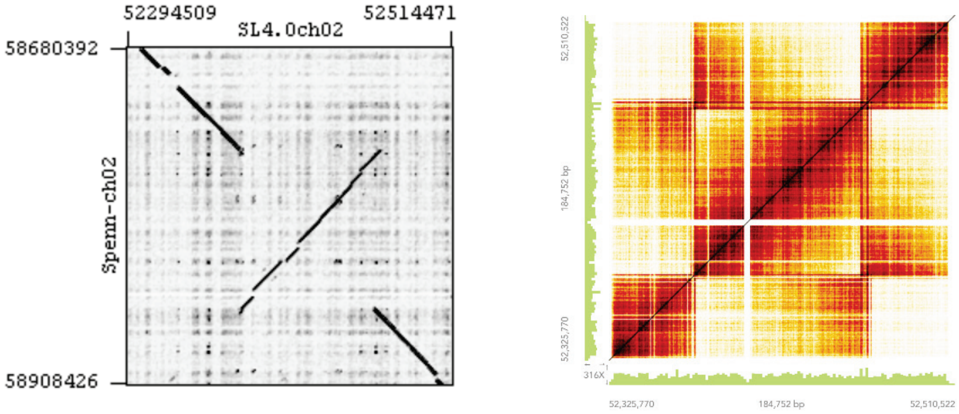

S4 Fig. **Validation of structural variants.** Examples of a (A) deletion and an (B) inversion that are validated by manual inspection. First, through dot plots between the assemblies of *S. lycopersicum c.v.* Heinz 1706 and *S. pennellii* genomes generated using *Gepard* (left). Second, through heatmap of overlapping barcodes between linked reads (10X Genomics) in the *S. pennellii* parental genome generated using *Loupe Browser*. The patterns in the top right and bottom right Figs characterize a deletion and an inversion, respectively.
