# Supplementary material for: A catalogue of recombination coldspots in interspecific tomato hybrids
Source: PLoS Genet. 2024 Jul 1;20(7):e1011336. doi: 10.1371/journal.pgen.1011336 (PMC11244794; doi:10.1371/journal.pgen.1011336)
Supplement: S5 Fig — (PDF) [file pgen.1011336.s010.pdf]

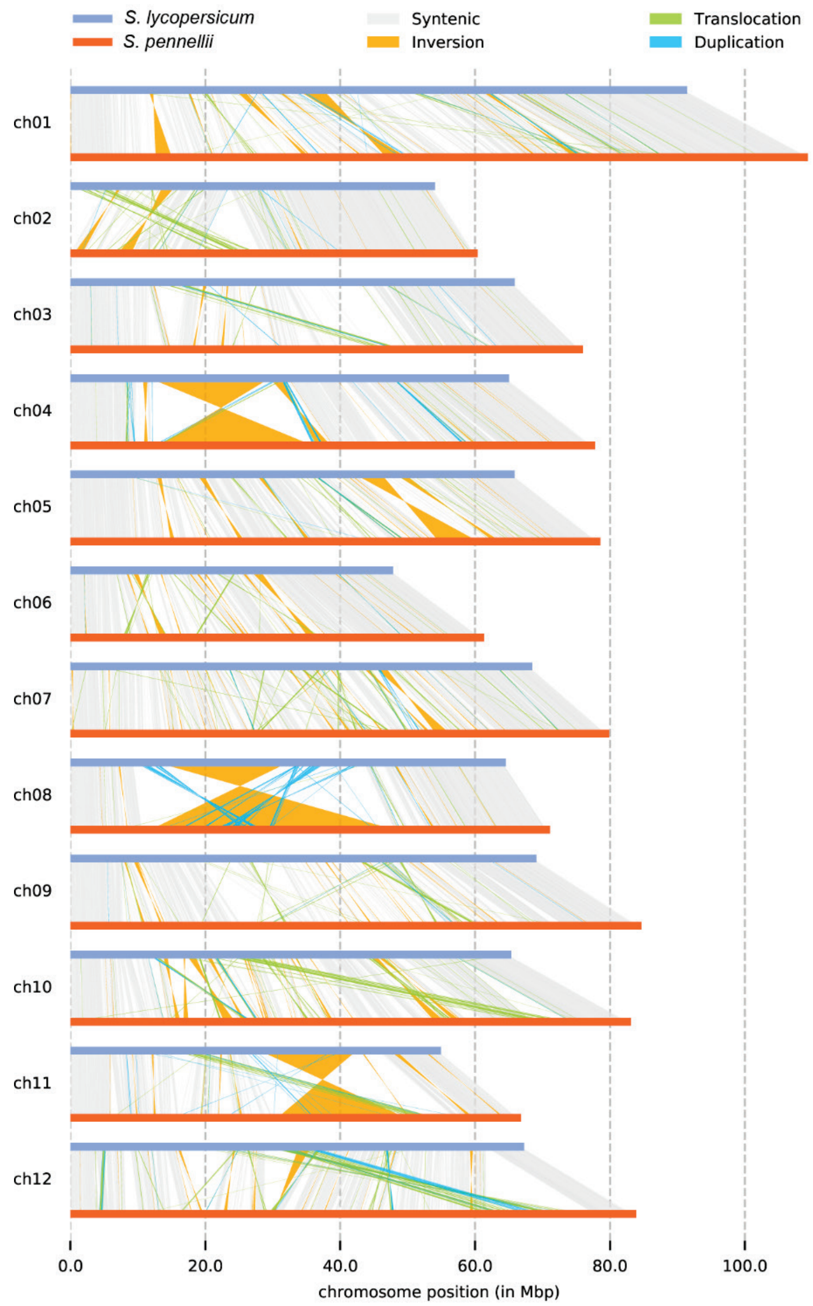

S5 Fig. **Parental genome alignment.** Alignment between the assemblies of *S. lycopersicum* and *S. pennellii* showing syntenic regions and rearrangements.
