# Supplementary material for: A catalogue of recombination coldspots in interspecific tomato hybrids
Source: PLoS Genet. 2024 Jul 1;20(7):e1011336. doi: 10.1371/journal.pgen.1011336 (PMC11244794; doi:10.1371/journal.pgen.1011336)
Supplement: S6 Fig — (PDF) [file pgen.1011336.s011.pdf]

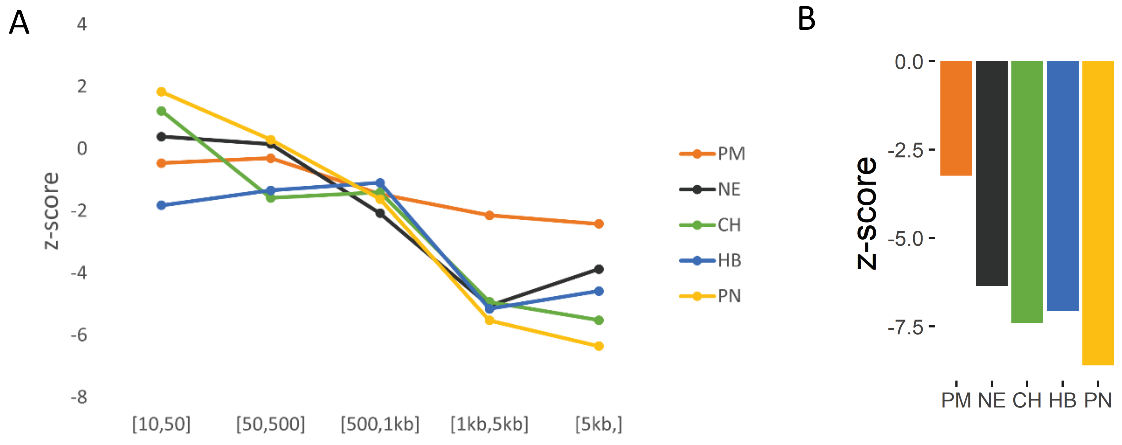

S6 Fig. **Suppression of COs in SVs.** A) To determine the relation between SV size and CO suppression, SVs are first binned according to size. Afterwards, per bin, the overlap of SVs and COs in the observed data was compared against the overlap in 10,000 permutation sets. Only bin [1kb,5kb] and [5kb,] have significantly fewer COs in SVs than expected by chance for all populations. B) Suppression of COs in SVs (size above 1kb) based on permutation test. The negative z-score means the overlap of COs in SV regions is lower than expected by chance.
