# Supplementary material for: A catalogue of recombination coldspots in interspecific tomato hybrids
Source: PLoS Genet. 2024 Jul 1;20(7):e1011336. doi: 10.1371/journal.pgen.1011336 (PMC11244794; doi:10.1371/journal.pgen.1011336)
Supplement: S7 Fig — (PDF) [file pgen.1011336.s012.pdf]

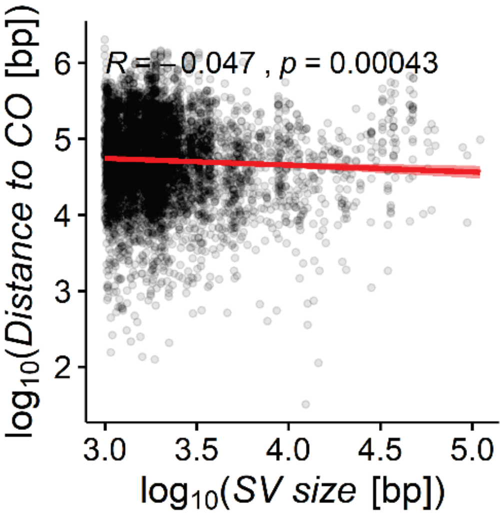


S7 Fig. **Distance of SVs to COs by size.** There is no association between SV size and the distance of the nearest CO.
