# Supplementary material for: A catalogue of recombination coldspots in interspecific tomato hybrids
Source: PLoS Genet. 2024 Jul 1;20(7):e1011336. doi: 10.1371/journal.pgen.1011336 (PMC11244794; doi:10.1371/journal.pgen.1011336)
Supplement: S8 Fig — (PDF) [file pgen.1011336.s013.pdf]

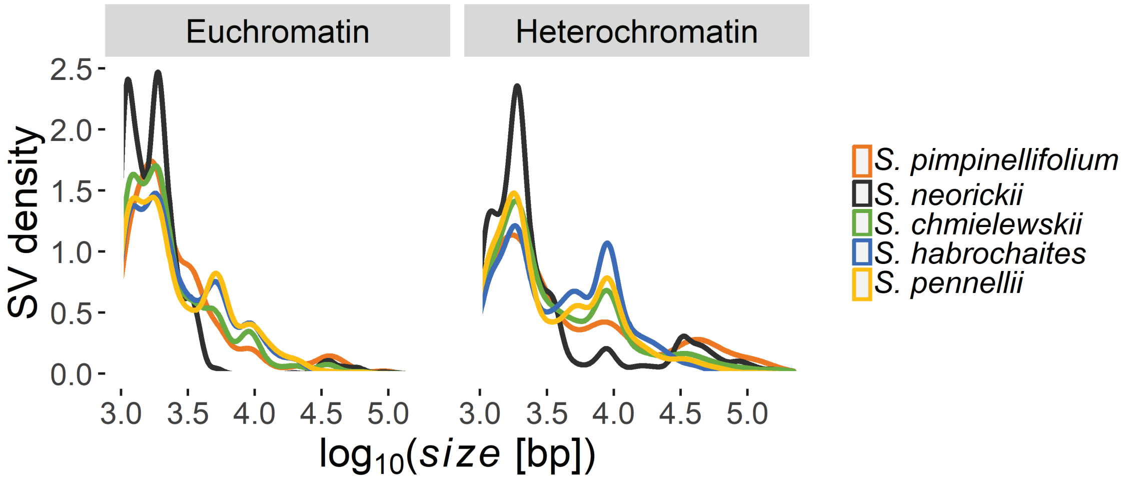

S8 Fig. **Sizes of structural variants.** Pericentric heterochromatin contains longer SVs in most wild genomes.
