# Supplementary material for: A catalogue of recombination coldspots in interspecific tomato hybrids
Source: PLoS Genet. 2024 Jul 1;20(7):e1011336. doi: 10.1371/journal.pgen.1011336 (PMC11244794; doi:10.1371/journal.pgen.1011336)
Supplement: S9 Fig — (PDF) [file pgen.1011336.s014.pdf]

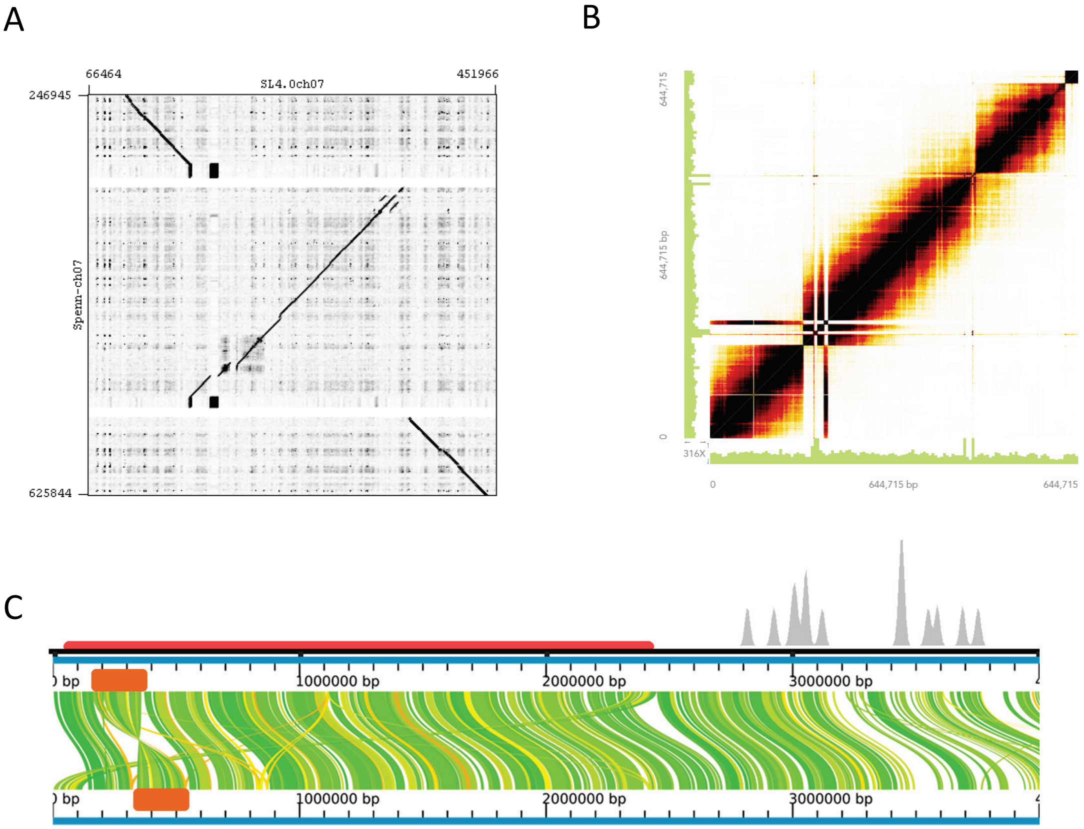

S9 Fig. **Inversion in chromosome 7 short arm.** A) A distal inversion between the short arm of *S. lycopersicum c.v.* Heinz 1706 and the *S.pennellii* assembly was visualized using a dot plot. B) Heatmap of overlapping barcodes between linked reads (10X Genomics) in the inversion region of *S. pennellii*. The figure was generated using *Loupe Browser*. C) Large inversion (orange block) and rearrangements within the PN coldspot (horizonal purple segment) of chromosome 7, short arm. CO density is indicated in grey at the top.
