# Supplementary material for: A catalogue of recombination coldspots in interspecific tomato hybrids
Source: PLoS Genet. 2024 Jul 1;20(7):e1011336. doi: 10.1371/journal.pgen.1011336 (PMC11244794; doi:10.1371/journal.pgen.1011336)
Supplement: S10 Fig — (PDF) [file pgen.1011336.s015.pdf]

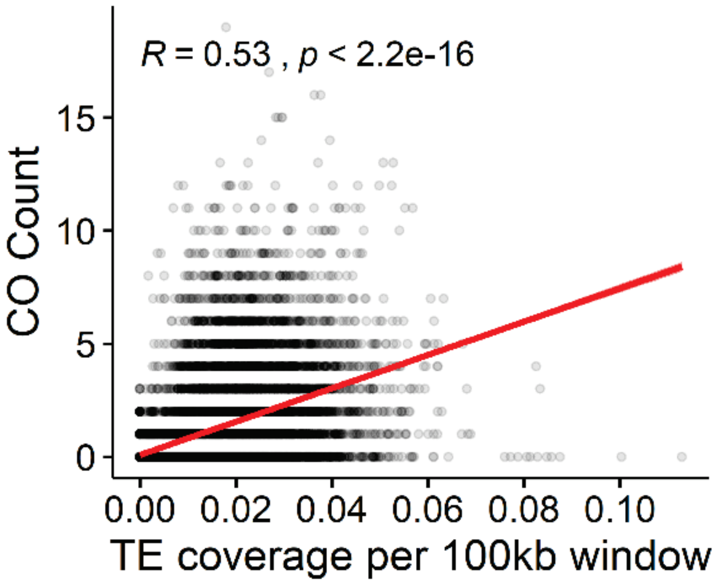

S10 Fig. **TE and CO correlation**. Spearman's rank correlation of crossover count and DNA transposons (*Stowaway* and *Tip100*) coverage in a sliding genome window. Each dot indicates a window.
