# Supplementary material for: A catalogue of recombination coldspots in interspecific tomato hybrids
Source: PLoS Genet. 2024 Jul 1;20(7):e1011336. doi: 10.1371/journal.pgen.1011336 (PMC11244794; doi:10.1371/journal.pgen.1011336)
Supplement: S11 Fig — (PDF) [file pgen.1011336.s016.pdf]

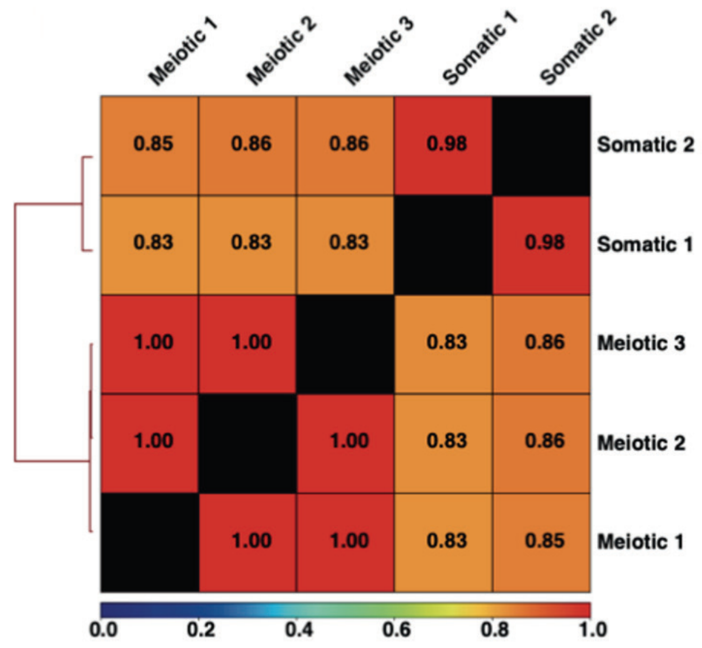

S11 Fig. **Pearson correlation of ACRs**. Comparison of read distribution over the genome between tissues and between biological replicates.
