# Supplementary material for: A catalogue of recombination coldspots in interspecific tomato hybrids
Source: PLoS Genet. 2024 Jul 1;20(7):e1011336. doi: 10.1371/journal.pgen.1011336 (PMC11244794; doi:10.1371/journal.pgen.1011336)
Supplement: S12 Fig — (PDF) [file pgen.1011336.s017.pdf]

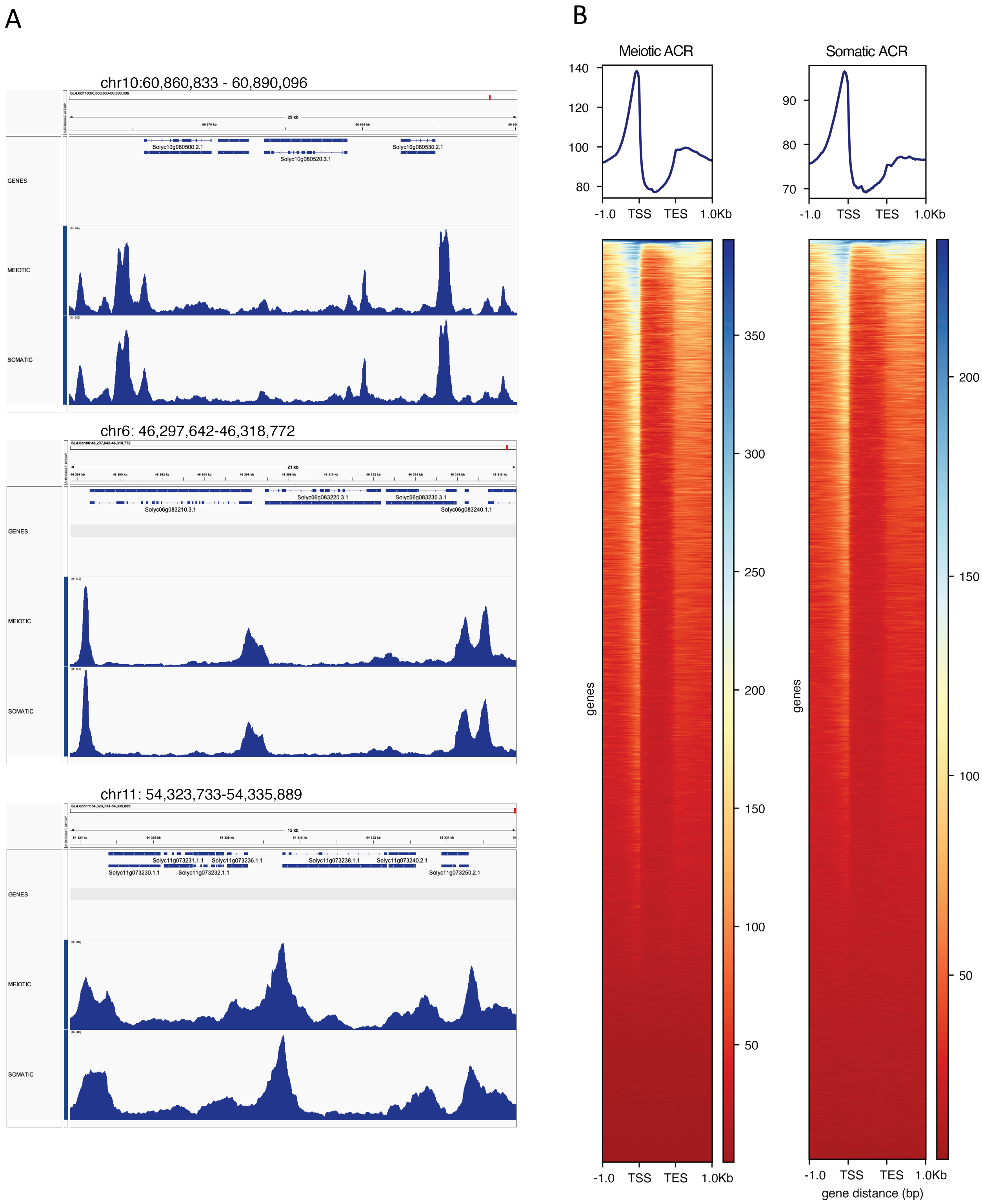

S12 Fig. **ATAC-seq peaks at transcription start sites (TSS)**. A) Representative snapshots of the integrative genome browser showing ATAC-seq peaks at transcription start sites (TSS). The meiotic and somatic tracks are group-scaled to have comparable y-axis. The meiotic tracks show the average signal of 3 technical replicates and the somatic tracks of 2 technical replicates. B) ATAC-seq coverage at *S. lycopersicum* annotated genes. Each row of the heatmap represents a gene. The color gradient shows the strength of the ATAC signal.
