# Supplementary material for: A catalogue of recombination coldspots in interspecific tomato hybrids
Source: PLoS Genet. 2024 Jul 1;20(7):e1011336. doi: 10.1371/journal.pgen.1011336 (PMC11244794; doi:10.1371/journal.pgen.1011336)
Supplement: S13 Fig — (PDF) [file pgen.1011336.s018.pdf]

A

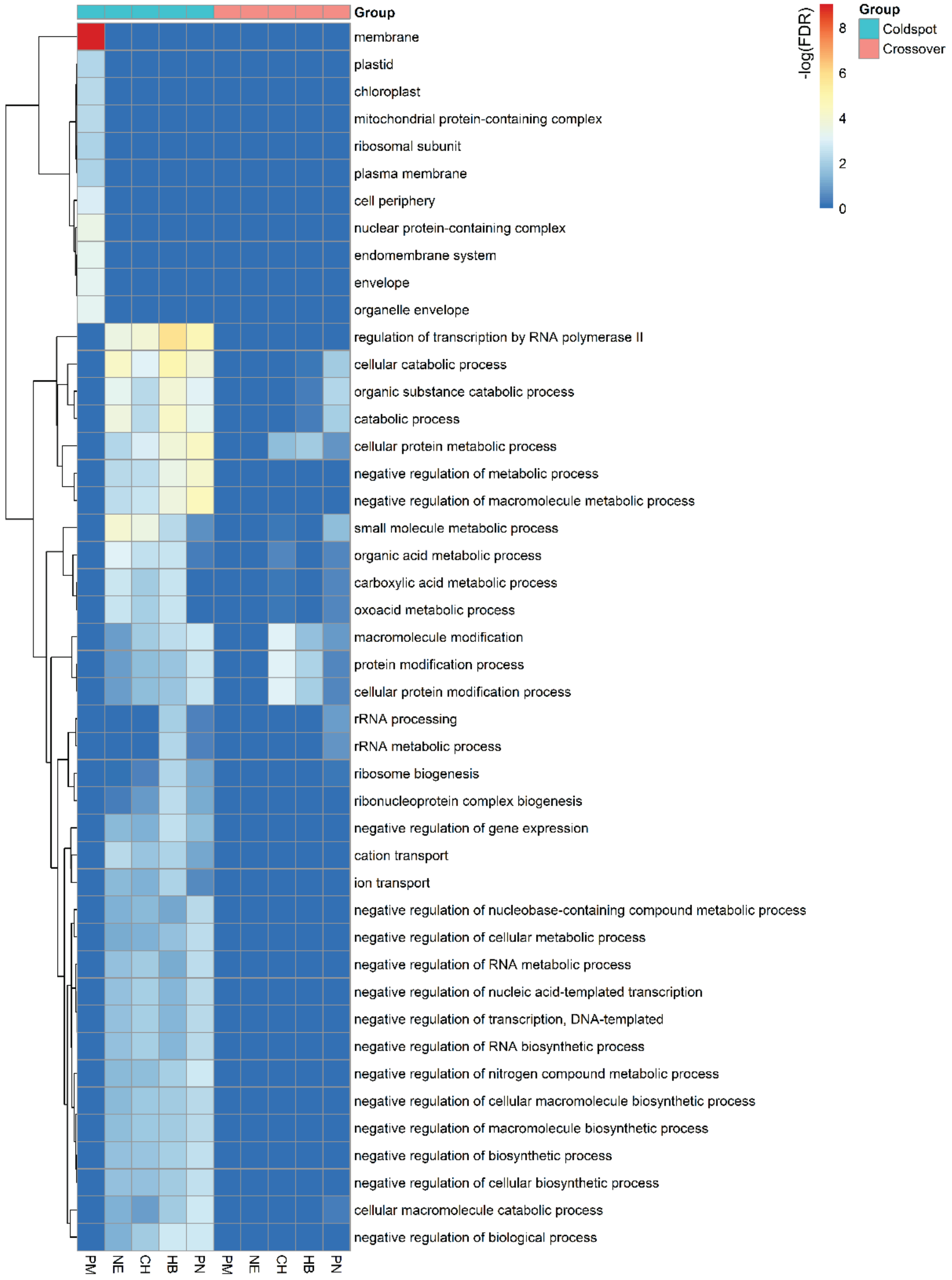

Group
membrane
plastid
chloroplast
mitochondrial protein-containing complex
ribosomal subunit
plasma membrane
cell periphery
nuclear protein-containing complex
endomembrane system
envelope
organelle envelope
regulation of transcription by RNA polymerase II
cellular catabolic process
organic substance catabolic process
catabolic process
cellular protein metabolic process
negative regulation of metabolic process
negative regulation of macromolecule metabolic process
small molecule metabolic process
organic acid metabolic process
carboxylic acid metabolic process
oxoacid metabolic process
macromolecule modification
protein modification process
cellular protein modification process
rRNA processing
rRNA metabolic process
ribosome biogenesis
ribonucleoprotein complex biogenesis
negative regulation of gene expression
cation transport
ion transport
negative regulation of nucleobase-containing compound metabolic process
negative regulation of cellular metabolic process
negative regulation of RNA metabolic process
negative regulation of nucleic acid-templated transcription
negative regulation of transcription, DNA-templated
negative regulation of RNA biosynthetic process
negative regulation of nitrogen compound metabolic process
negative regulation of cellular macromolecule biosynthetic process
negative regulation of macromolecule biosynthetic process
negative regulation of biosynthetic process
negative regulation of cellular biosynthetic process
cellular macromolecule catabolic process
negative regulation of biological process
PM
NE
CH
HB
PN
PM
NE
CH
HB
PN
-log(FDR)
8
6
4
2
0
Group
Coldspot
Crossover

B

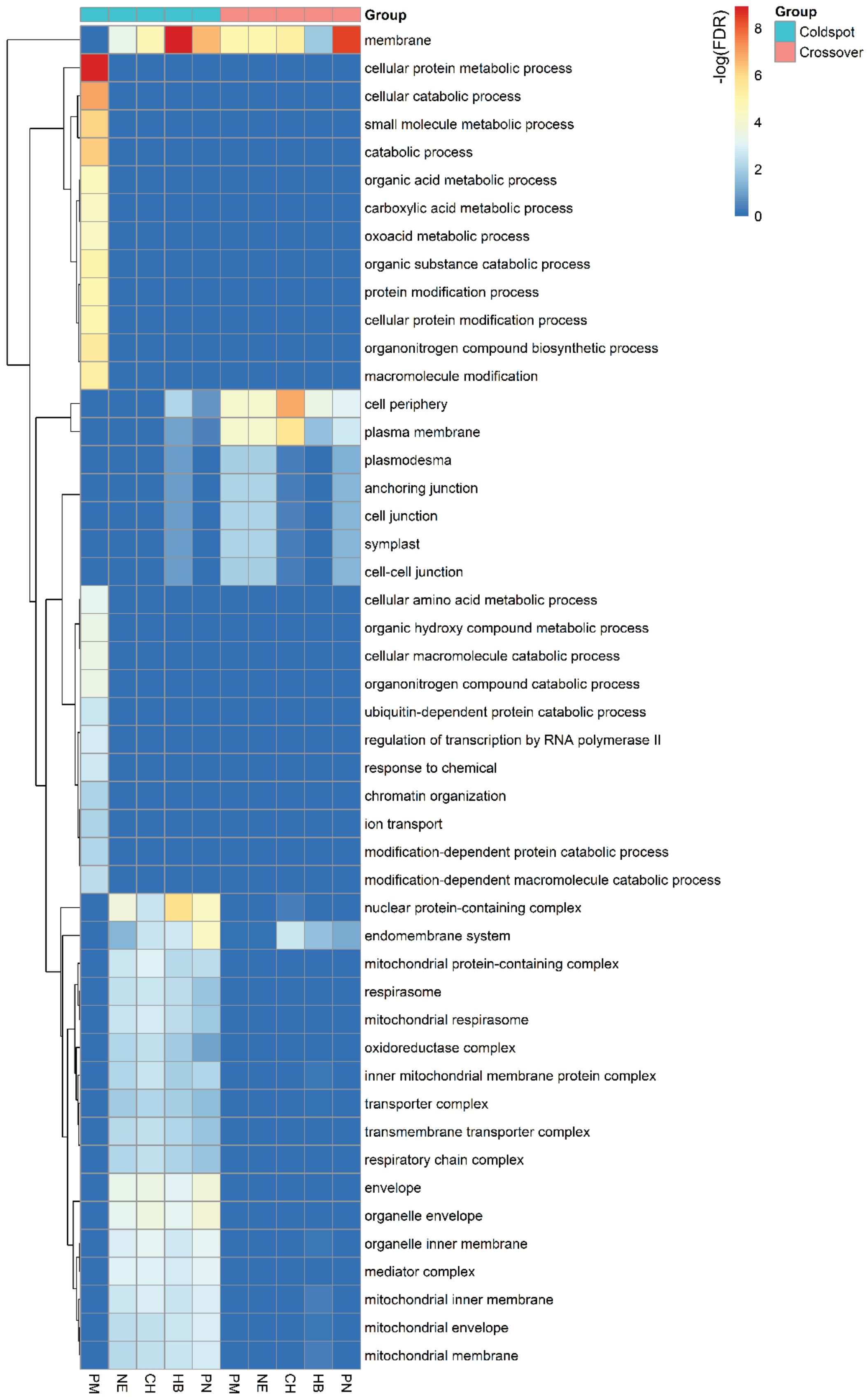
Group
-log(FDR)
8
6
4
2
0
Group
Coldspot
Crossover
membrane
cellular protein metabolic process
cellular catabolic process
small molecule metabolic process
catabolic process
organic acid metabolic process
carboxylic acid metabolic process
oxoacid metabolic process
organic substance catabolic process
protein modification process
cellular protein modification process
organonitrogen compound biosynthetic process
macromolecule modification
cell periphery
plasma membrane
plasmodesma
anchoring junction
cell junction
symplast
cell-cell junction
cellular amino acid metabolic process
organic hydroxy compound metabolic process
cellular macromolecule catabolic process
organonitrogen compound catabolic process
ubiquitin-dependent protein catabolic process
regulation of transcription by RNA polymerase II
response to chemical
chromatin organization
ion transport
modification-dependent protein catabolic process
modification-dependent macromolecule catabolic process
nuclear protein-containing complex
endomembrane system
mitochondrial protein-containing complex
respirasome
mitochondrial respirasome
oxidoreductase complex
inner mitochondrial membrane protein complex
transporter complex
transmembrane transporter complex
respiratory chain complex
envelope
organelle envelope
organelle inner membrane
mediator complex
mitochondrial inner membrane
mitochondrial envelope
mitochondrial membrane
PM
NE
CH
HB
PN
PM
NE
CH
HB
PN

C

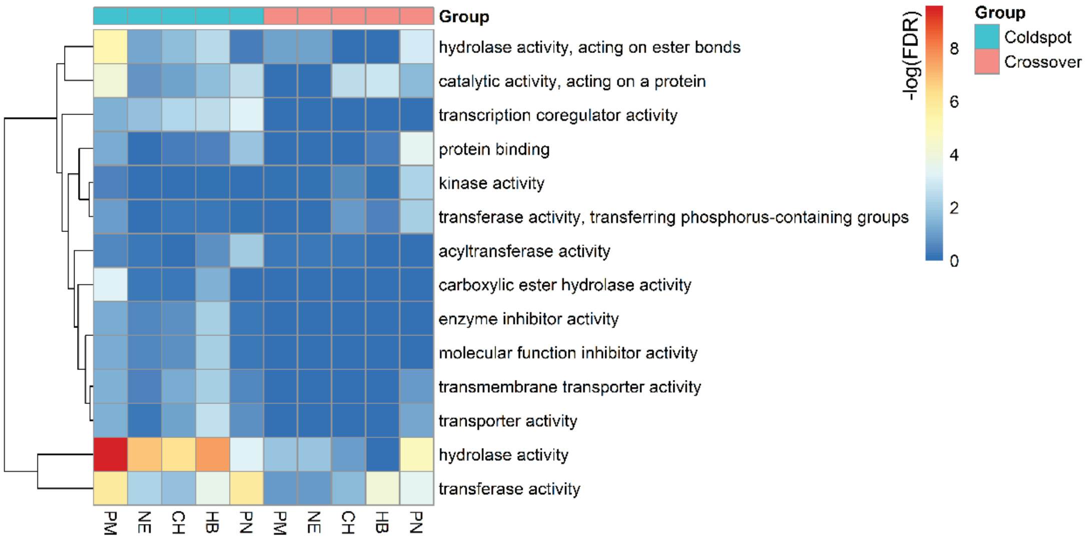

S13 Fig. **Functional enrichment in coldspot and CO regions**. The minimum fold enrichment is 1.5. We separately reported the overrepresented terms by category: biological process (A), cellular location (B) and molecular function (C).
