# Supplementary material for: A catalogue of recombination coldspots in interspecific tomato hybrids
Source: PLoS Genet. 2024 Jul 1;20(7):e1011336. doi: 10.1371/journal.pgen.1011336 (PMC11244794; doi:10.1371/journal.pgen.1011336)
Supplement: S14 Fig — (PDF) [file pgen.1011336.s019.pdf]

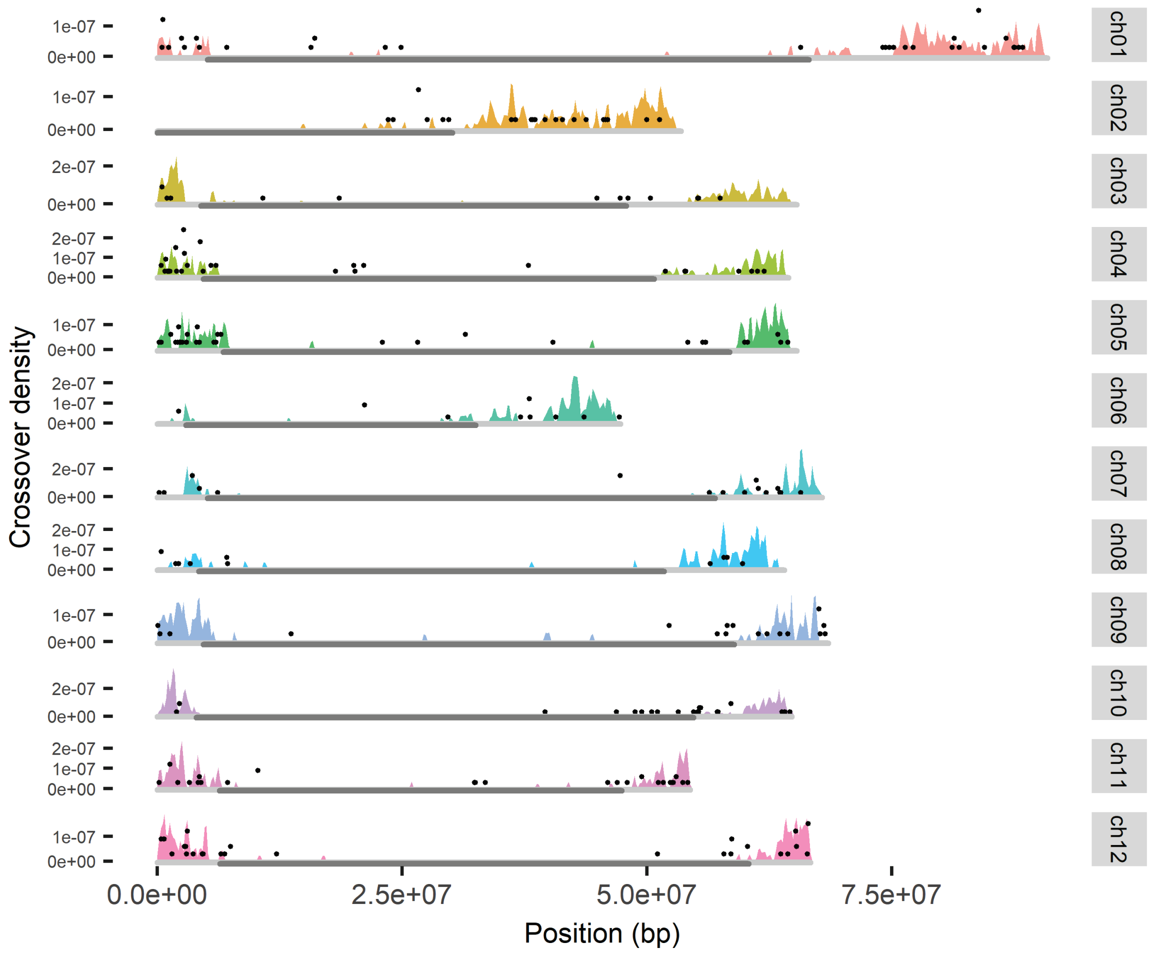

S14 Fig. **Resistance genes across the tomato genome**. The black dots representing the frequency of R genes is plotted with the recombination landscape of the *S. lycopersicum* x *S. pennellii* hybrid.
