# Supplementary material for: A catalogue of recombination coldspots in interspecific tomato hybrids
Source: PLoS Genet. 2024 Jul 1;20(7):e1011336. doi: 10.1371/journal.pgen.1011336 (PMC11244794; doi:10.1371/journal.pgen.1011336)
Supplement: S15 Fig — (PDF) [file pgen.1011336.s020.pdf]

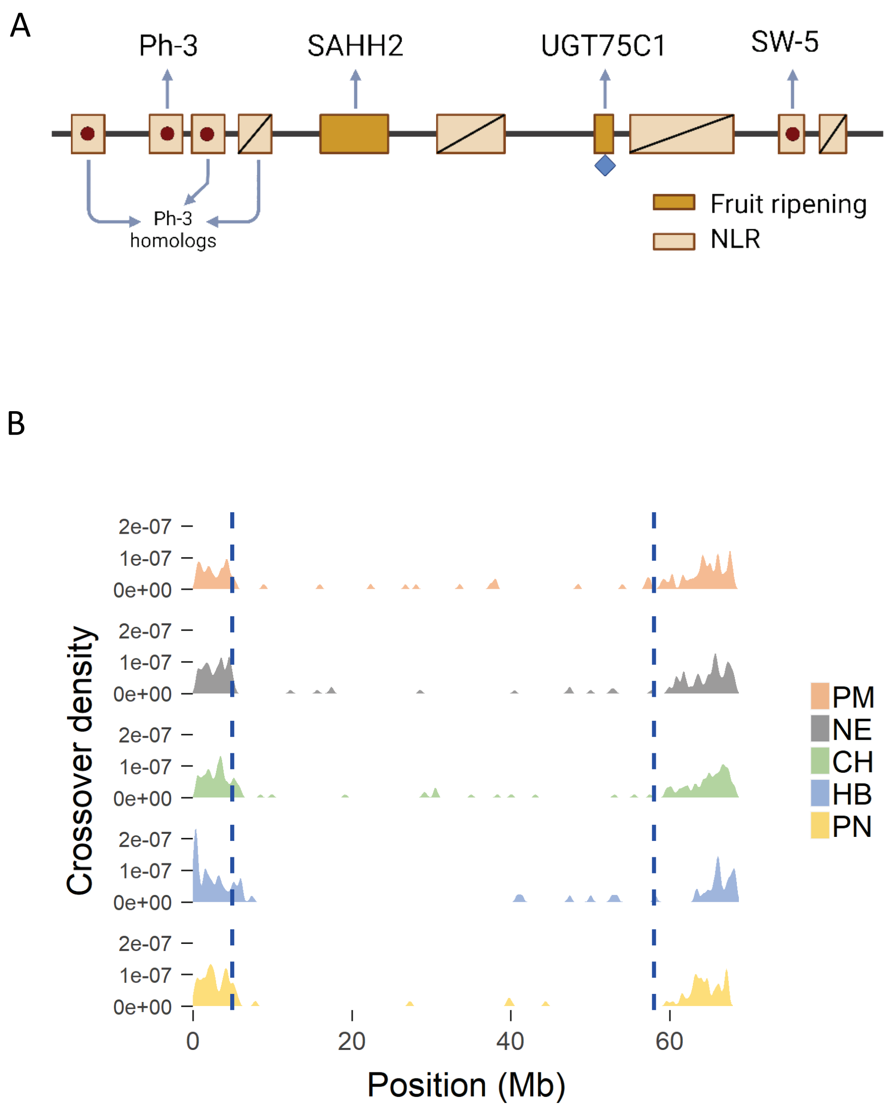

S15 Fig. **Unfavorable linkage.** A) Coldspot in PN chromosome 9 containing genes associated with resistance and agronomic traits. Red dots and diagonal lines indicate missense mutations and frameshift mutations, respectively. Blue diamond indicates the differential expression between the parental genomes. B) Rare recombination events in the *Tm-2* introgression, which span the region between the two blue lines, in chromosome 9.
