# Supplementary material for: A catalogue of recombination coldspots in interspecific tomato hybrids
Source: PLoS Genet. 2024 Jul 1;20(7):e1011336. doi: 10.1371/journal.pgen.1011336 (PMC11244794; doi:10.1371/journal.pgen.1011336)
Supplement: S16 Fig — (PDF) [file pgen.1011336.s021.pdf]

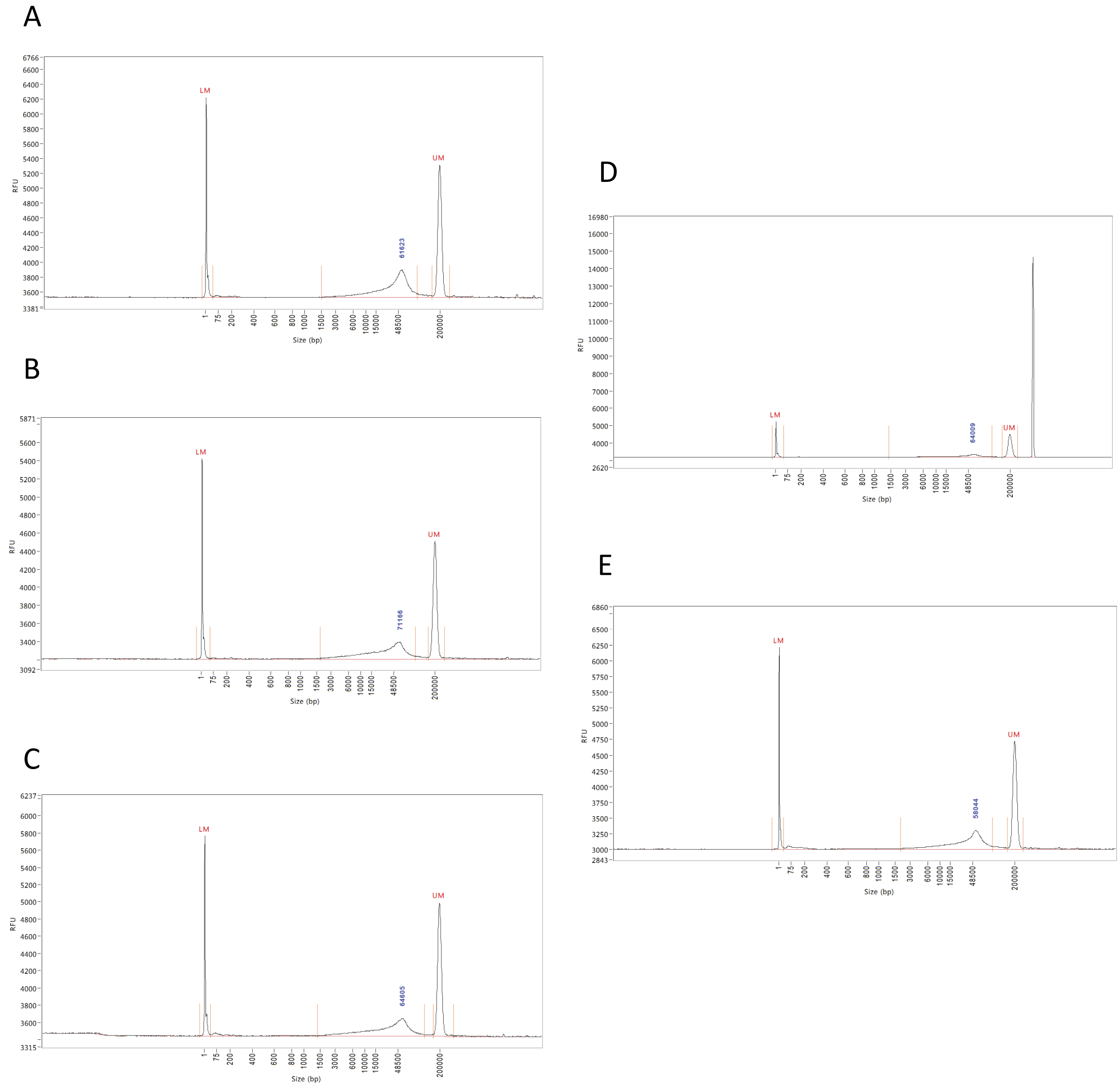


S16 Fig. **DNA molecule lengths generated using Fragment Analyzer**. A) *S. pimpinellifolium.* B) *S. neorickii.* C) *S. chimielewskii.* D) *S. habrochaites.* E) *S. pennellii.*
