# Supplementary material for: A catalogue of recombination coldspots in interspecific tomato hybrids
Source: PLoS Genet. 2024 Jul 1;20(7):e1011336. doi: 10.1371/journal.pgen.1011336 (PMC11244794; doi:10.1371/journal.pgen.1011336)
Supplement: S17 Fig — (PDF) [file pgen.1011336.s022.pdf]

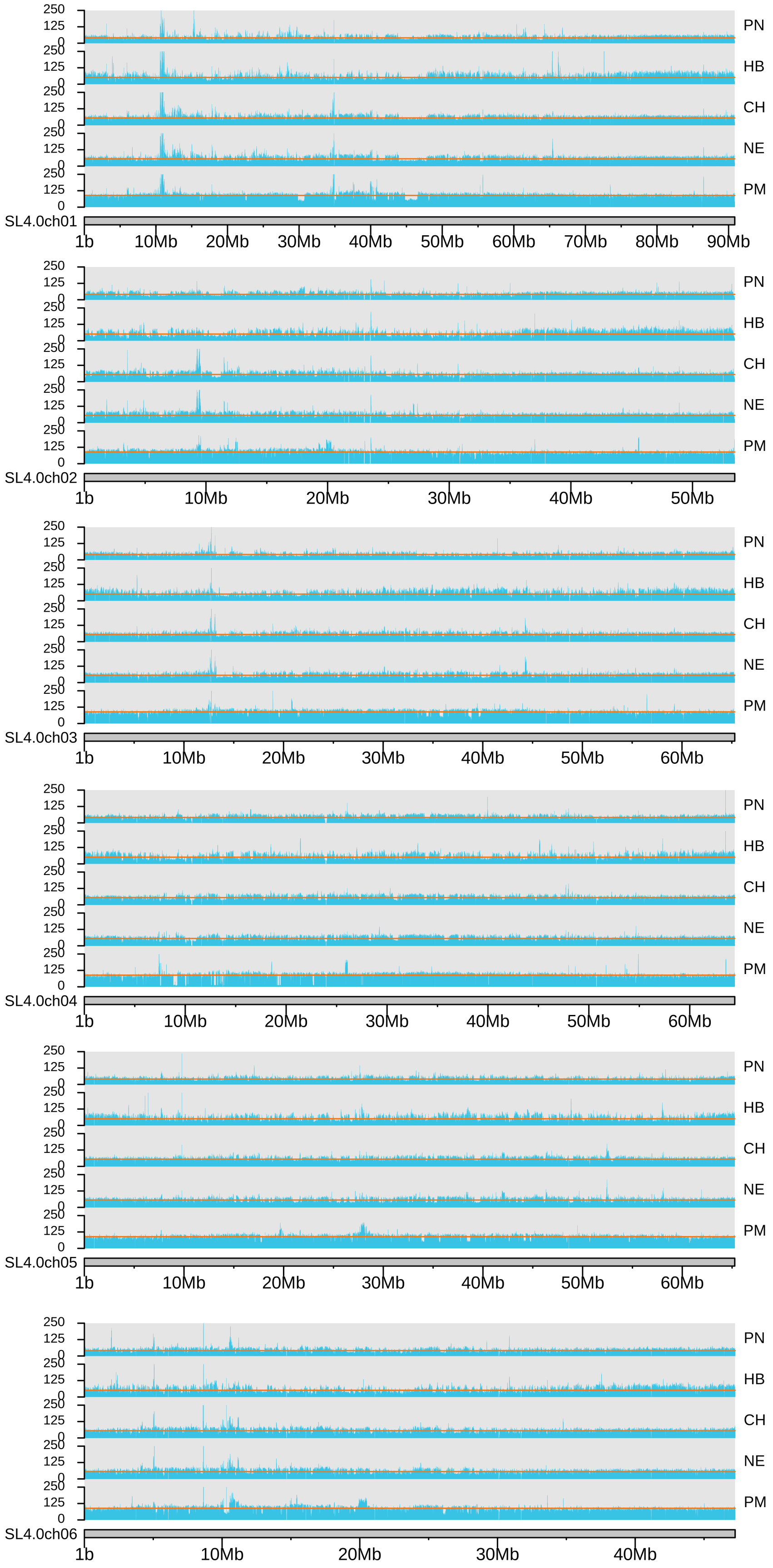

250
125
0
PN
HB
CH
NE
PM
SL4.0ch01
1b
10Mb
20Mb
30Mb
40Mb
50Mb
60Mb
70Mb
80Mb
90Mb
SL4.0ch02
1b
10Mb
20Mb
30Mb
40Mb
50Mb
SL4.0ch03
1b
10Mb
20Mb
30Mb
40Mb
50Mb
60Mb
SL4.0ch04
1b
10Mb
20Mb
30Mb
40Mb
50Mb
60Mb
SL4.0ch05
1b
10Mb
20Mb
30Mb
40Mb
50Mb
60Mb
SL4.0ch06
1b
10Mb
20Mb
30Mb
40Mb

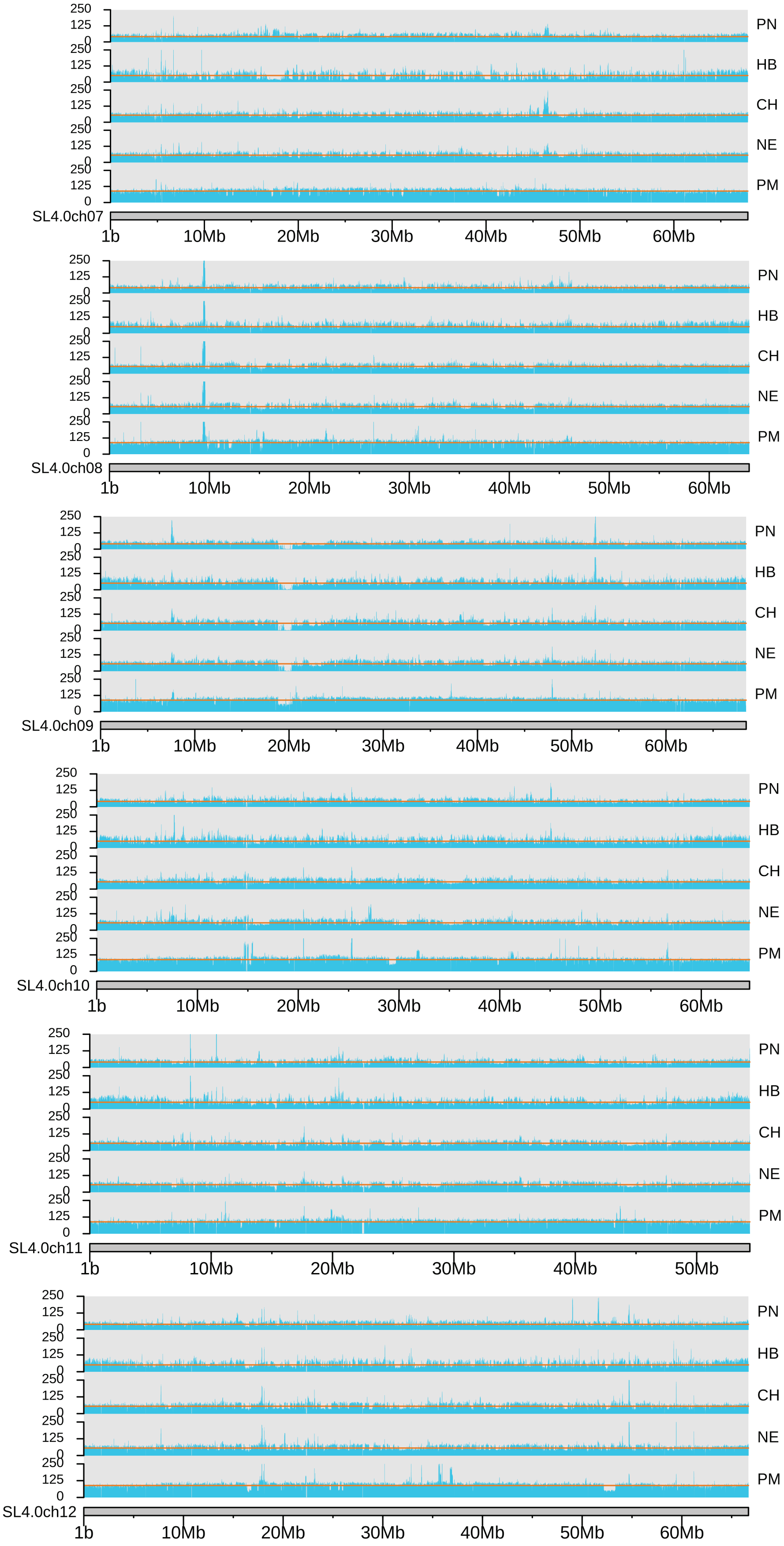

S17 Fig. **Linked-read coverage along the chromosomes.** Shown in y-axis is the median read coverage in 10-kb sliding window with 2-kb step size. The orange line indicates the genome-wide median coverage.
