# Supplementary material for: A catalogue of recombination coldspots in interspecific tomato hybrids
Source: PLoS Genet. 2024 Jul 1;20(7):e1011336. doi: 10.1371/journal.pgen.1011336 (PMC11244794; doi:10.1371/journal.pgen.1011336)
Supplement: S18 Fig — (PDF) [file pgen.1011336.s023.pdf]

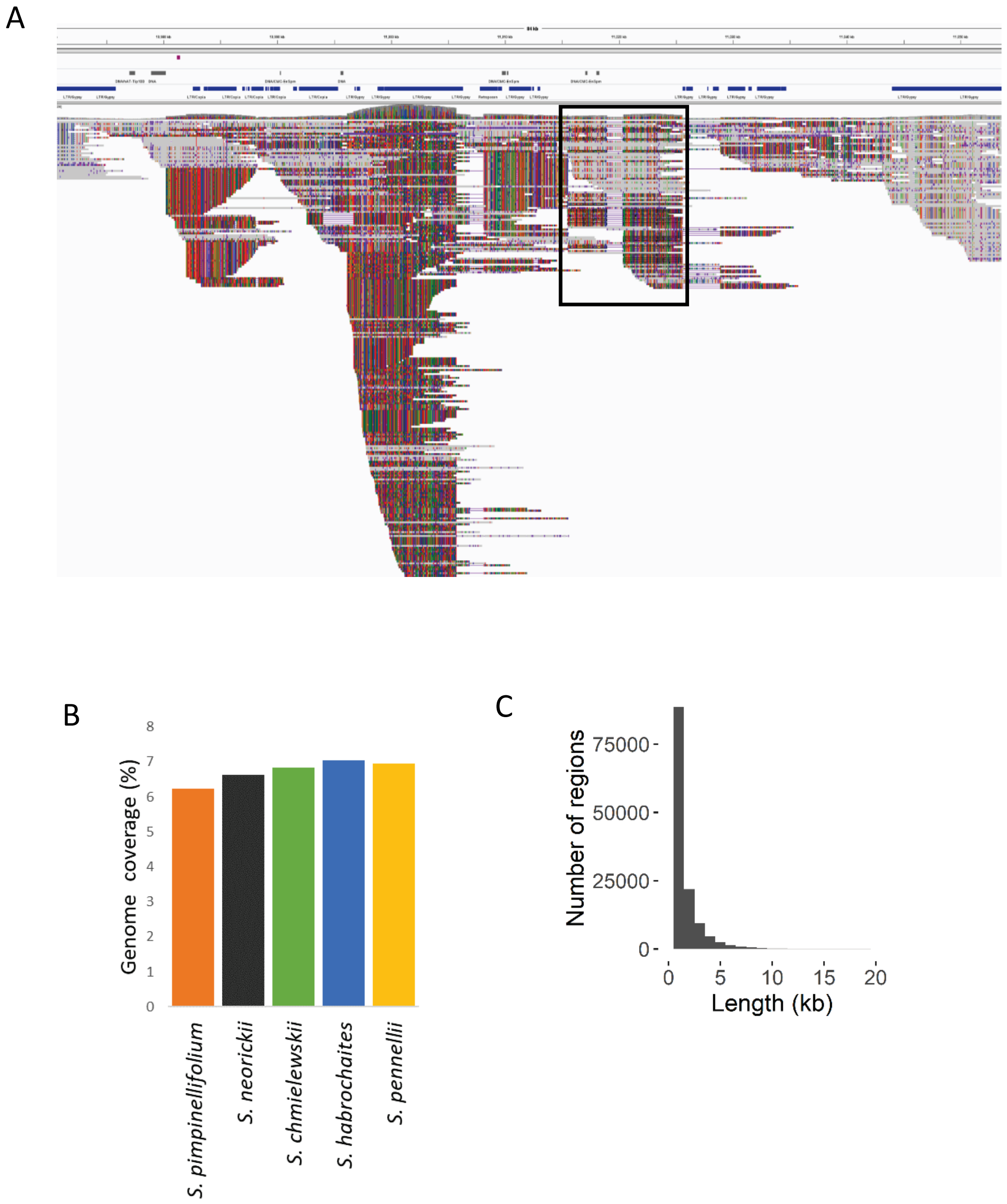

S18 Fig. **False positive hotspots in pericentromeres**. A) Regions with excessive levels of heterozygosity and read coverage (gray lines) causing false positive crossovers (black box). The blue lines represent copies of transposable elements. Possibly, these regions are collapsed genomic segments in the reference genome or part of a copy number variation. B) The coverage and C) length distribution of these regions.
