# Supplementary material for: A catalogue of recombination coldspots in interspecific tomato hybrids
Source: PLoS Genet. 2024 Jul 1;20(7):e1011336. doi: 10.1371/journal.pgen.1011336 (PMC11244794; doi:10.1371/journal.pgen.1011336)
Supplement: S19 Fig — (PDF) [file pgen.1011336.s024.pdf]

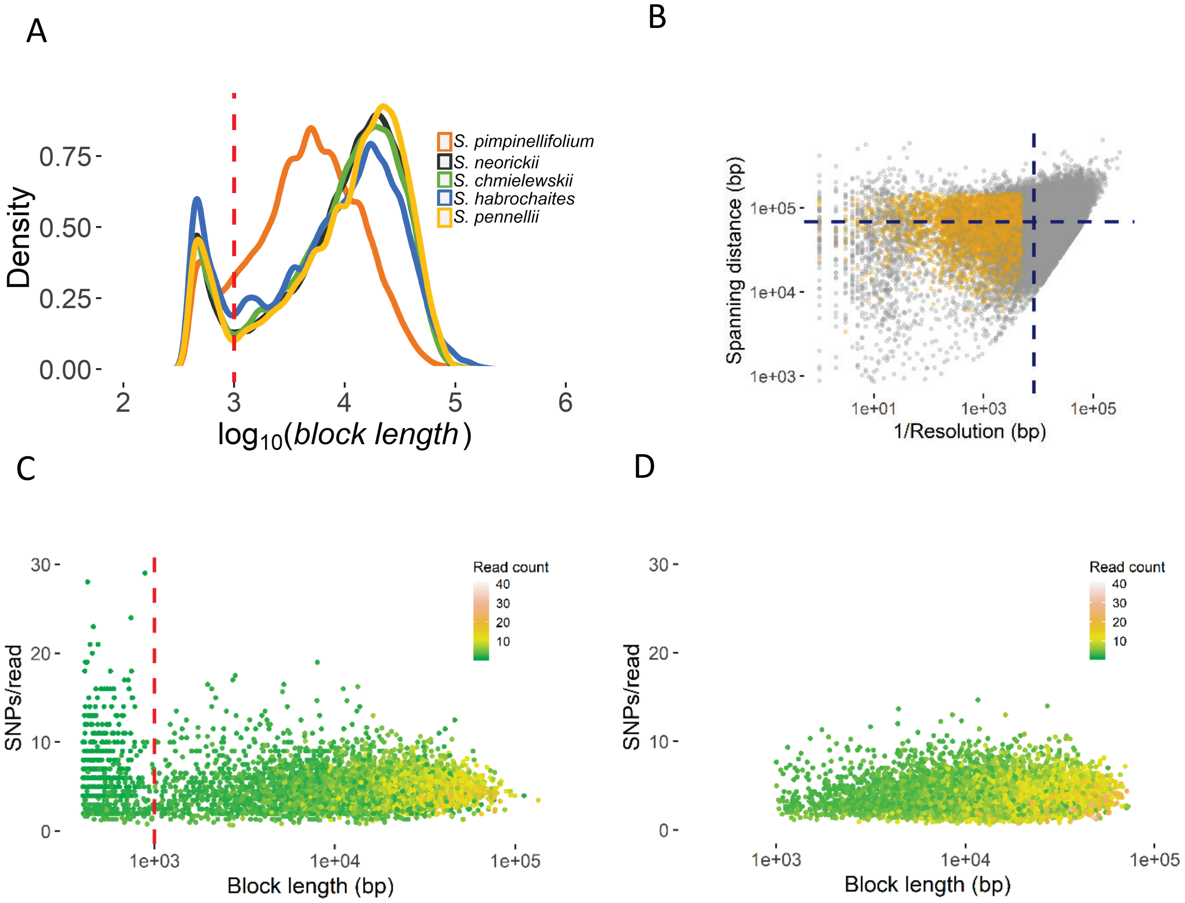


S19 Fig. **Filtering criteria on recombination molecules**. A) A lower limit on the size of a haplotype block (marked by a red vertical line) is set based on the distribution of all block sizes across all crosses. B) Distribution of resolution and spanning distance of each recombinant molecule. The yellow dots represent COs that passed the filtering. The blue lines mark the average spanning distance and resolution. C) Ratio of SNP and read count per haplotype block as a function of block length. Haplotype blocks with sizes below 1kb are supported by few reads with high SNP density which may result from mismapped reads, further supporting the cut-off for haplotype block size. D) Final set of COs that passed all filtering constraints.
