# Supplementary material for: A catalogue of recombination coldspots in interspecific tomato hybrids
Source: PLoS Genet. 2024 Jul 1;20(7):e1011336. doi: 10.1371/journal.pgen.1011336 (PMC11244794; doi:10.1371/journal.pgen.1011336)
Supplement: S20 Fig — (PDF) [file pgen.1011336.s025.pdf]

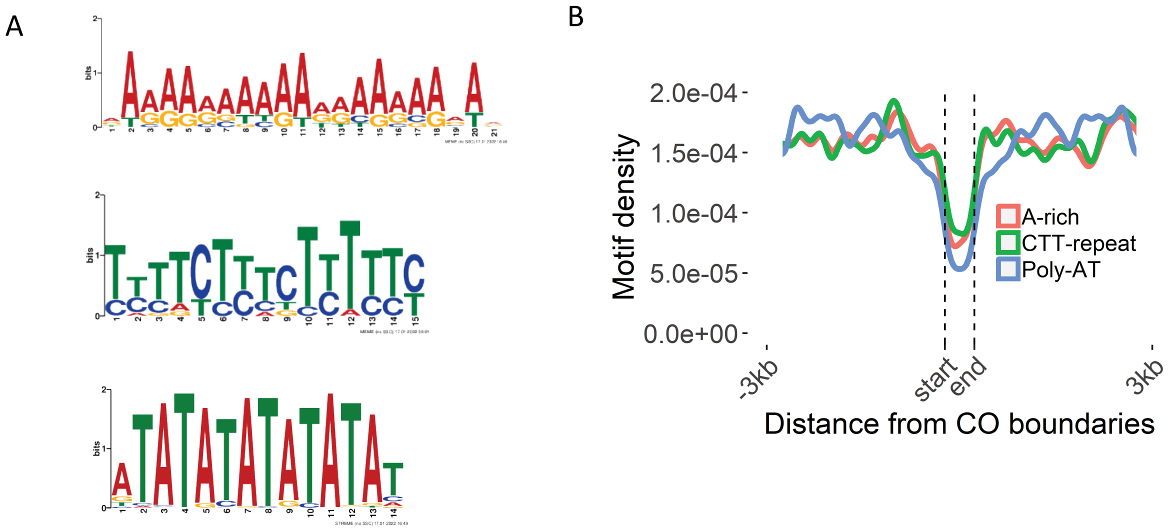

S20 Fig. **Overrepresented motifs**. A) Motifs found within and flanking CO sites. Of 1,267 COs with resolution of at least 0.002, only 8-28% have the motif within the CO sites while the rest contain multiple copies of the motifs in the flanking regions. B) Distribution of the motifs within and around high-resolution COs. Start and end mark the boundaries of the CO regions.
